# Supplementary material for: Network state changes in sensory thalamus represent learned outcomes
Source: Nat Commun. 2024 Sep 7;15:7830. doi: 10.1038/s41467-024-51868-8 (PMC11380690; doi:10.1038/s41467-024-51868-8)
Supplement: Supplementary file 1 — Supplementary Information [file 41467_2024_51868_MOESM1_ESM.pdf]

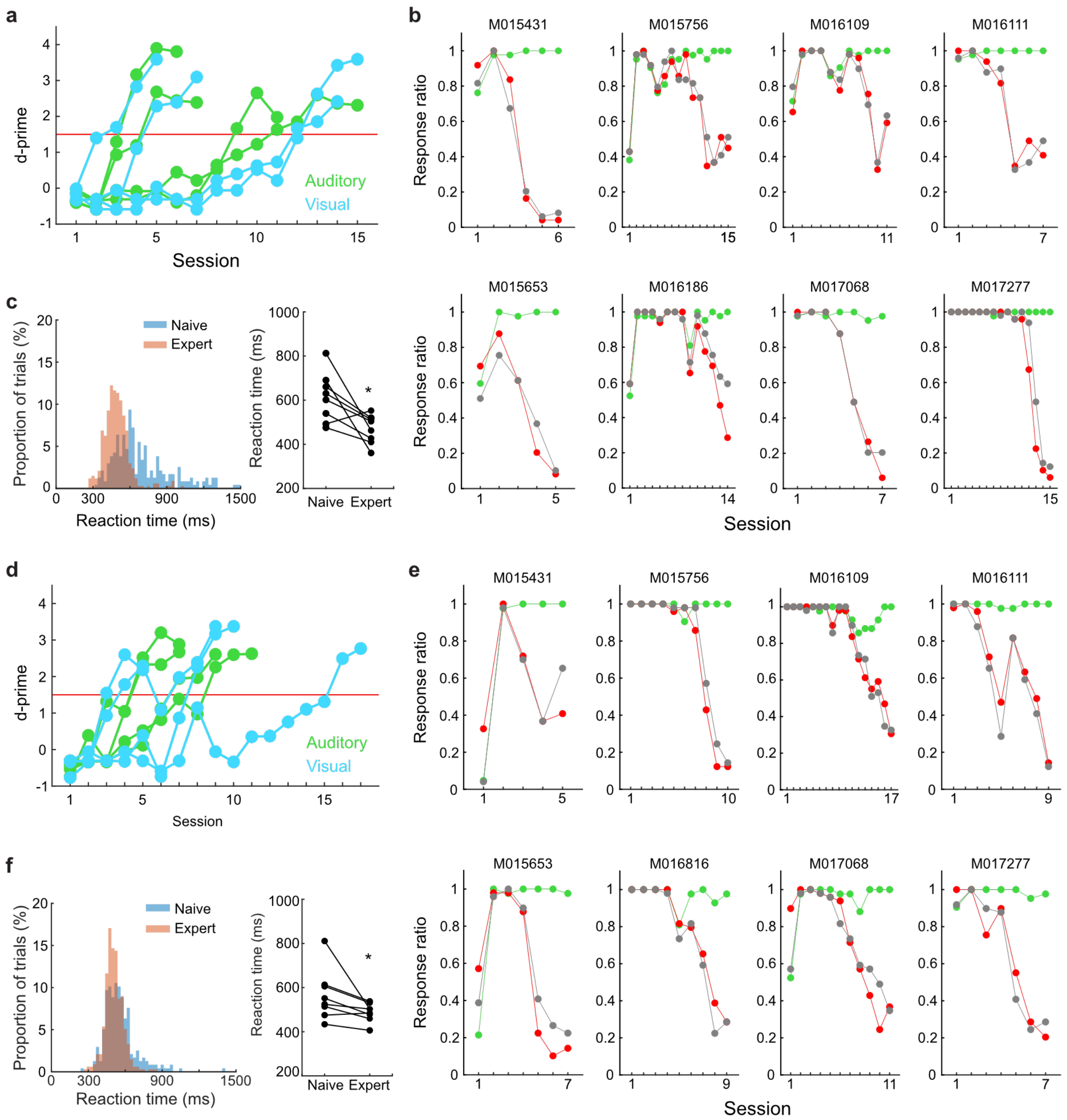

Supplementary Figure 1 | Task performance of individual mice during the sensory Go/Nogo task. (**a-c**) Task performance of individual mice and Hit reaction time (RT) during initial learning. (**a**) d-prime transition of all mice. (**b**) Task performance of individual mice. Top row: Auditory-reward group. Bottom row: Visual-reward group. (**c**) Left: Hit reaction time pooled across all mice in the naive and expert phases. Right: Transition of median Hit reaction time. Each dot represents an individual mouse. Reaction times in the expert phase were shorter than in the naive phase (signed-rank test). The first behavioral session in the naive phase and the session with the highest d-prime in the expert phase were used for the reaction time analysis. (**d-f**) Task performance of individual mice and Hit reaction time in the reversal learning. Panel configurations are the same as **a-c**. Reaction times in the expert phase were shorter than in the naive phase (signed-rank test). * represents the statistical significance of *p*<0.05.

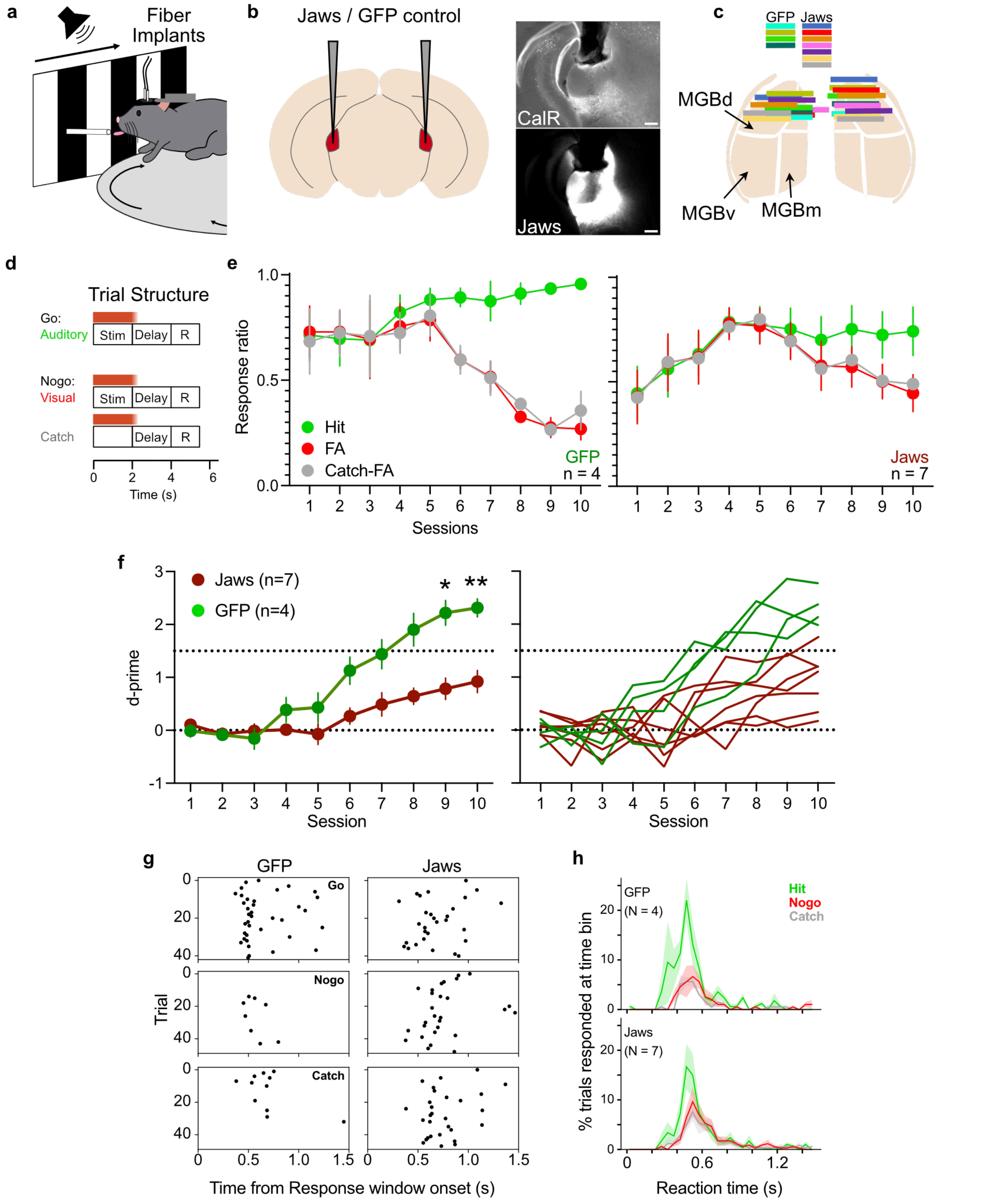

Supplementary Figure 2 | MGB activity is required for sensory learning. (a) Schematic of the behavioral setup for optogenetic inhibition. **(b)** Left: Schematic of the bilateral AAV injections into MGB. AAV expressing inhibitory opsin (Jaws) was bilaterally injected into MGB for the opsin-positive group. AAV expressing GFP was bilaterally injected into MGB for the control group. Right: Confocal images of the fiber implantation location. Right top: Calretinin (CalR) staining. Right bottom: Jaws expression in MGB. Scale bar: 200 μm. **(c)** Fiber implantation locations in MGB. Each line represents a fiber front and each color represents an individual mouse. **(d)** Trial structure of the behavioral task is similar to the calcium imaging experiments. Optogenetic perturbation was carried out in all trial types during the stimulus period (2 s). For details see Methods. **(e)** Response ratios of the GFP control (left, N = 4) and Jaws-positive group (right, N = 7) to all trial types over the training sessions. **(f)** Transition of d-prime in optogenetic inhibition experiments. Left: Mean of d-prime in the GFP-control and Jaws-positive groups over training sessions (Mean +/- SEM). d-primes of the Jaws-positive group were significantly lower than those of the control group at the end of the training (Day 9 & 10, p = 0.0230 and p = 0.0067 respectively, Two-way ANOVA with Sidak's multiple comparison test). Right: The transition of d-prime from all mice. Each line represents d-prime values of individual mice. **(g)** Lick raster plot from one example mouse in the GFP-control (left) and Jaws-positive group (right). For each trial type, trial 0 is the first trial. **(h)** Distribution of the first lick latencies for each trial type for the GFP (top) and Jaws-positive (bottom) groups. Bin size: 50 ms.

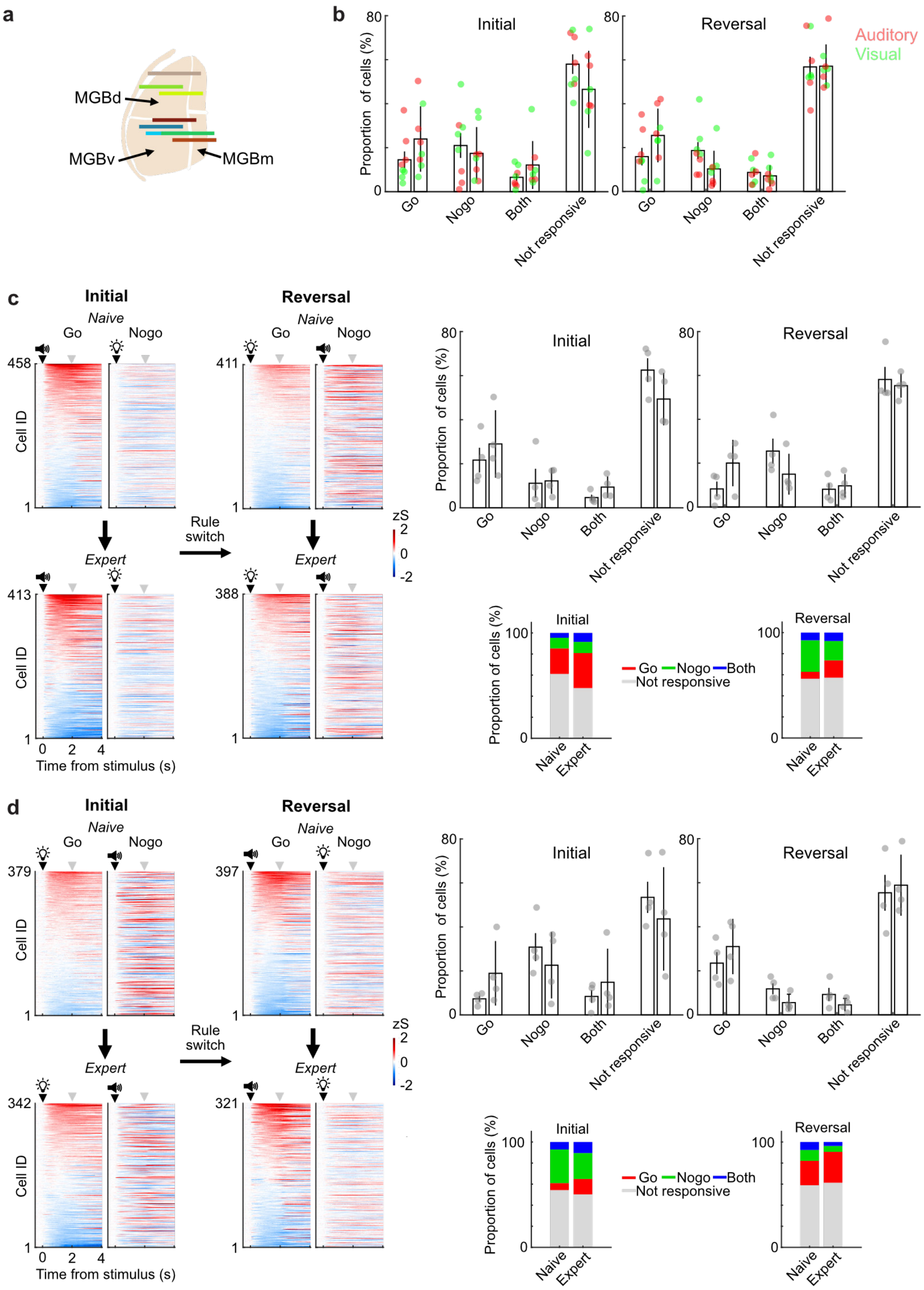

Supplementary Figure 3 | Activity of individual cells and the proportion of stimulus-responsive cells in auditory and visual reward groups. (**a**) GRIN lens front and putative field of view (FOV) from all mice (N = 8 mice). Each line represents the lens front and the putative FOVs of the two-photon image. For the 1.0 mm lens, the FOV is approximately 330 μm x 330 μm. For the 0.6 mm lens, the FOV is approximately 400 μm x 400 μm. Due to the distortion of the image through the GRIN lens, these FOV values do not necessarily reflect the actual size of the brain image. (**b**) The proportion of stimulus-responsive cells from all mice (N = 8 mice). (**c**) Mean individual cell responses and the proportion of stimulus-responsive cells from the Auditory-reward (initial learning) → Visual reward (reversal learning) group. Left: Heat maps of the mean individual cell activities in Go and Nogo trials of a single representative session during initial learning (n = 458 cells in the naive phase, n = 411 cells in the expert phase cells from 4 mice) and reversal learning (n = 413 cells in the naïve phase, n = 388 cells in the expert phase from 4 mice). Cells were sorted by the averaged response amplitude during the stimulus presentation in Go trials. Cell IDs are matched across Go and Nogo trials in each learning phase. Black and gray triangles represent the stimulus and delay period onset, respectively. Right top: The proportion of stimulus-responsive cells in initial and reversal learning. Right bottom: Stacked-bar charts of the stimulus-responsive cells from naive to expert phase in the initial and reversal learning. The proportions of stimulus responsive cells was altered from the naive to the expert phase across learning in both initial and reversal learning (p < 0.01, chi-square test, in both initial and reversal learning). (**d**) Mean individual cell responses and the proportion of stimulus-responsive cells from Visual-reward (initial learning) → Auditory reward (reversal learning) group. The figure configurations are the same as (**c**). The proportions of stimulus responsive cells were altered from the naive to the expert phase across learning in both initial and reversal learning (p<0.01 for Initial and p<0.05 reversal learning, after Bonferroni correction, chi-square test).

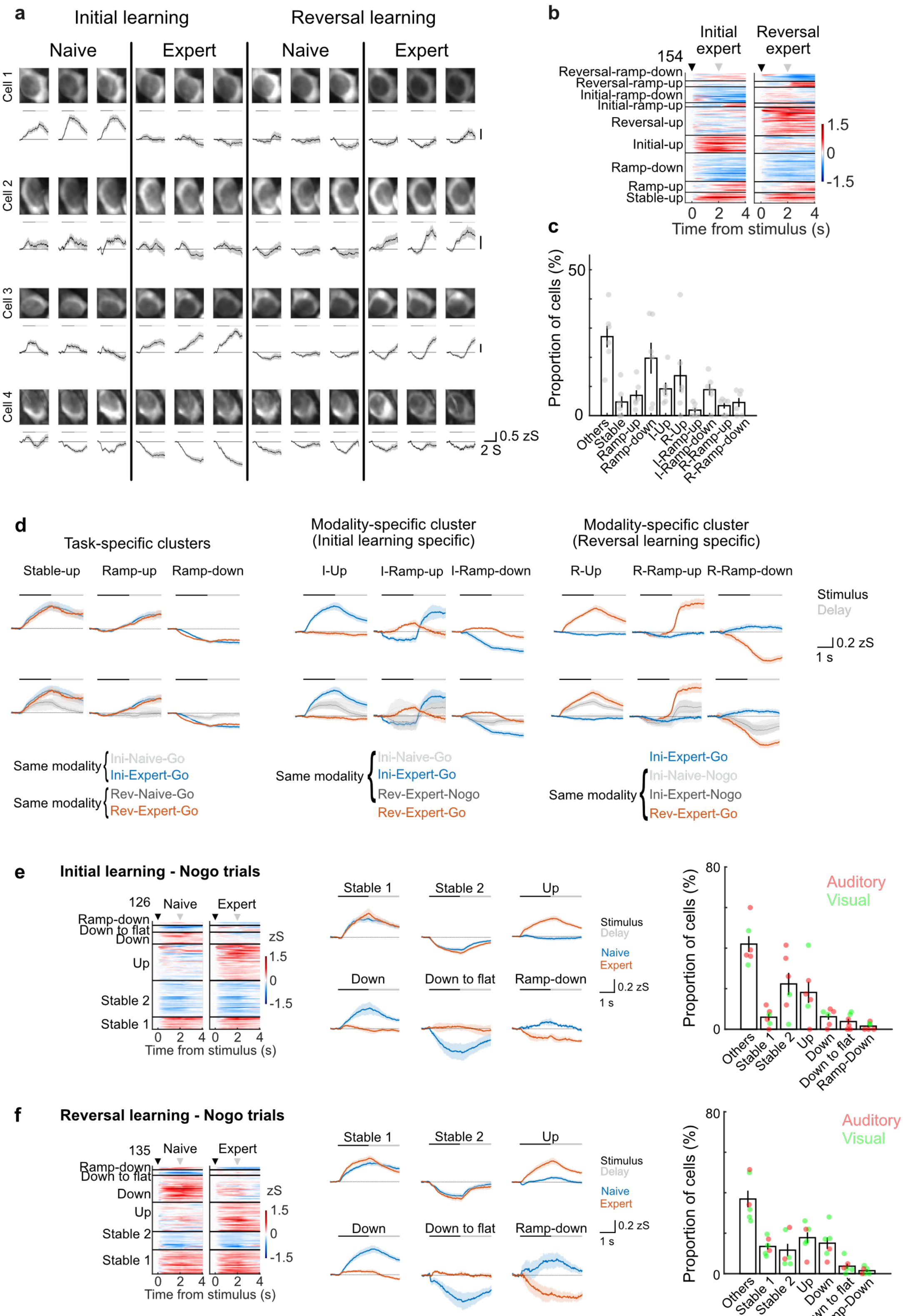

Supplementary Figure 4 | Task-specific and modality-specific clusters in Go trials and functional clusters in Nogo trials.

(**a**) Example of longitudinally-tracked MGB neurons and their calcium traces across learning in one mouse (n = 4 cells). (**b-d**) Functional subgroups with task and modality-specific learning plasticity in the two expert phases in both initial and reversal learning. (**b**) Heat maps of single cell activities in the two expert phases (N = 6 mice, n = 154 cells). (**c**) Proportion of cells in each functional cluster. Each dot represents the data from an individual mouse. (**d**) Average calcium traces (mean +/- SEM) of the functional subgroups shown in (**b**). Top row: Average calcium traces in the two expert phases. Left: Task-specific clusters. Middle: Modality-specific clusters showing the plasticity in initial learning. Right: Modality-specific clusters showing plasticity in reversal learning. Bottom row: Average calcium traces of the two expert phases with additional traces of other conditions with the same sensory modality. The additional traces were overlaid to show how reward learning modulated the neuronal activity within the same sensory modality. The figure configuration from Left to Right is the same as the top row. (**e**) Functional subgroups of MGB neurons in Nogo trials in initial learning. Left: Heat maps of single cell activities in the naive and expert phases in initial learning (N = 6 mice, n = 126 cells). Middle: Average calcium traces (mean +/- SEM) of the functional subgroups. Right: Proportion of cells in each cluster. Each dot represents the data from the individual mouse. Red and green dots represent the types of stimulus-reward condition. (**f**) Functional subgroups of MGB neurons in Nogo trials in reversal learning (N = 6 mice, n = 135 cells). The figure structures are the same as (**e**).

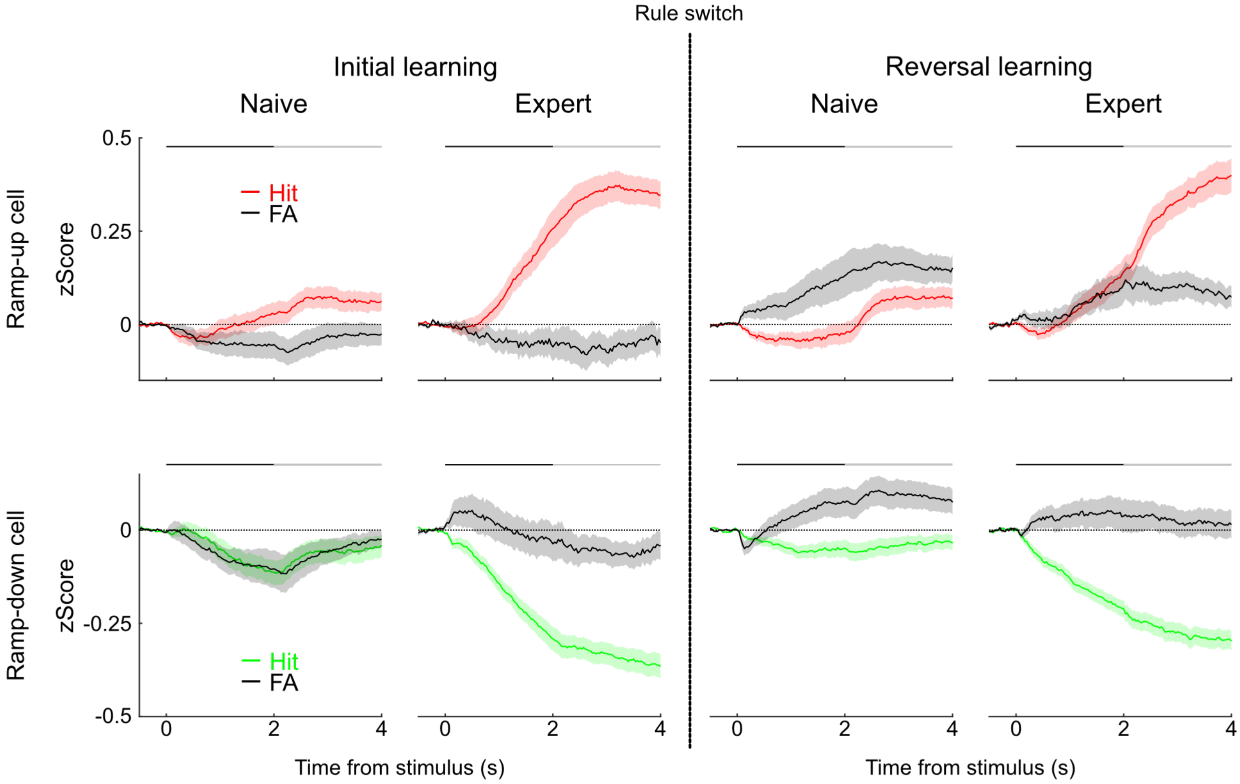

Supplementary Fig. 5 | Activities of ramp-up and ramp-down cells in Hit and FA trials. Both ramp-up and ramp-down cells showed their unique activity patterns in Hit trials. In FA trials, their activity patterns are distinct from Hit trials. Ramp-up neurons showed increased activities in FA trials in the naive phase of reversal learning. This could be due to reward anticipation in Nogo trials, which were previously Go trials in initial learning. Black and gray lines in each graph represent stimulus (2 s) and delay period (2 s), respectively.

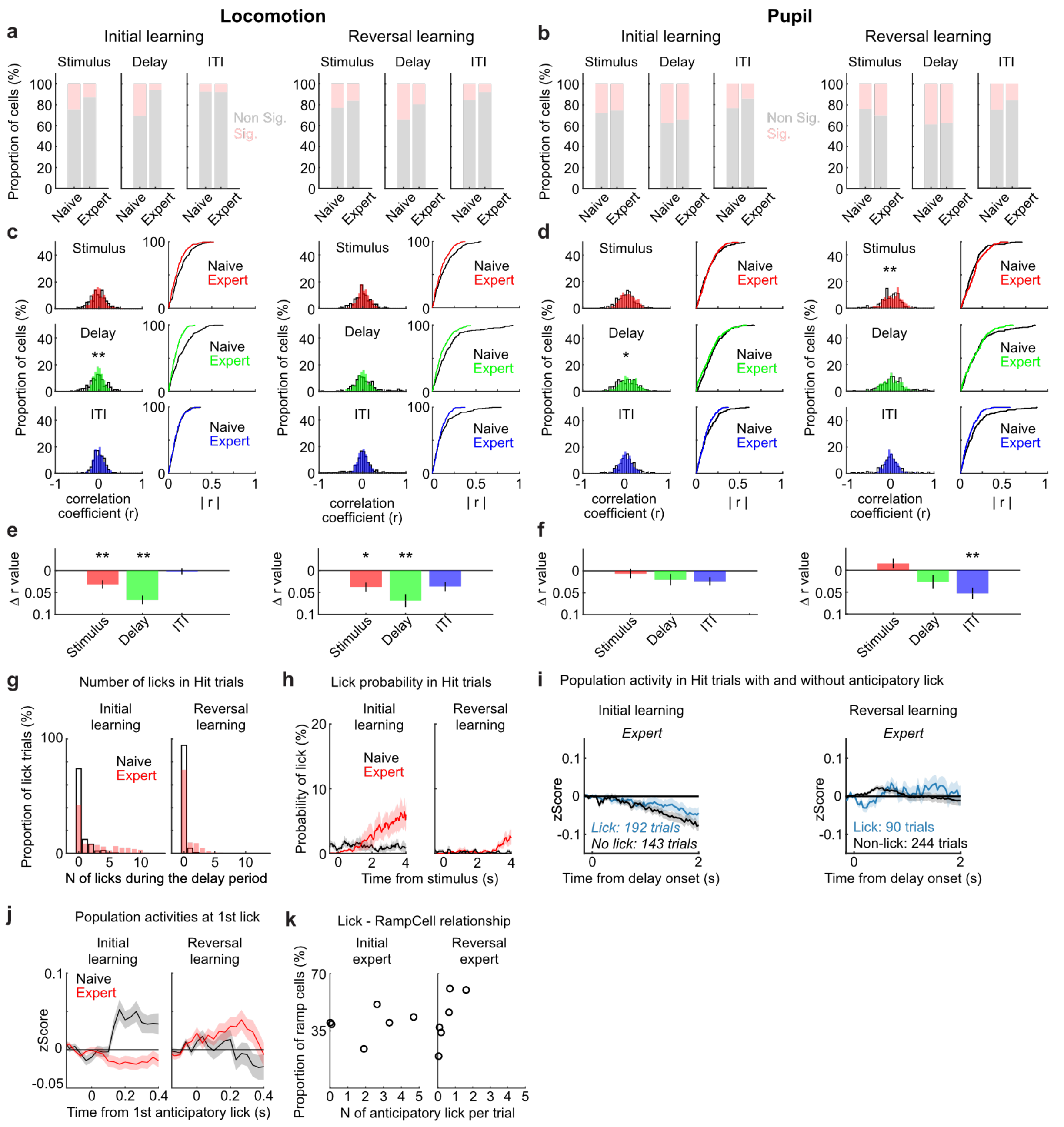

Supplementary Figure 6 | Behavioral variables are not the main source of MGB plasticity. (**a, c and e**) Correlation analysis of Ca²+ trace and locomotion in Hit trials in initial and reversal learning (N = 6 mice, n = 210 cells). Longitudinally tracked cells were used for this analysis. (**a**) Left: Proportion of the significantly correlated cells in each time window (Stimulus, Delay and ITI periods) in initial (Left) and reversal learning (Right). (**c**) Distributions of locomotion - Ca²+ correlation between the naive (black) and expert phases (color) in initial (Left) and reversal learning (Right) from all cells. Top: Probability density function during the stimulus period. Middle: Probability density function during the delay period. Bottom: Probability density function during the ITI period. ITI periods is 2 s before the stimulus presentation. (**e**) Change in the magnitude of correlation calculated as the difference of the absolute r value for each MGB neuron between naive and expert phases (Mean ± SEM). (**b, d and f**) Correlation analysis of Ca²+ trace and pupil size in Hit trials in initial and reversal learning (N = 6 mice, n = 210 cells). Panel configurations are the same as **a, c and e** for locomotion analysis. (**g**) The proportion of Hit trials with anticipatory licking during the delay period across all mice (N = 8 mice). The number of trials with anticipatory licking increased from naive to expert phases in both initial and reversal learning ($p <$ 0.01 for initial and reversal learning, after Bonferroni correction, chi-square test). (**h**) Probability of anticipatory licking at each time bin (Mean ± SEM) in Hit trials from all mice (N = 8 mice). The probability of anticipatory lick was calculated at each time bin across Hit trials for each mouse, then averaged across all mice. (**i**) Average population activity of MGB neurons (Mean ± SEM) during the delay period in Hit trials with and without anticipatory licking across all mice (N = 8 mice). Left: Population activity in the initial expert phase (Lick trial = 192 trials and n = 755 cells, non-lick trial = 143 trials and n = 755 cells), Right: Population activity in the reversal expert phase (Lick trial = 90 trials and n = 637 cells, non-lick trial = 244 trials and n = 709 cells). (**j**) Average population activity of MGB neurons (Mean ± SEM) aligned on the first anticipatory lick onset during the delay period. Left: Average population activity of MGB neurons in initial learning (61 trial, n = 749 cells for naive, 192 trials, n = 755 cells for expert). Right: Average population activity in reversal learning (13 trials, n = 604 cells for naive, 90 trials, n = 637 cells for expert). (**k**) The proportion of ramping cells (ramp-up and ramp-down cells) found in k-means analysis as a function of the mean of the number of anticipatory licks during the delay period per trial in Hit trials. * and ** in the figures represent statistical significance after Bonferroni correction. *, p<0.05, **, p<0.01.

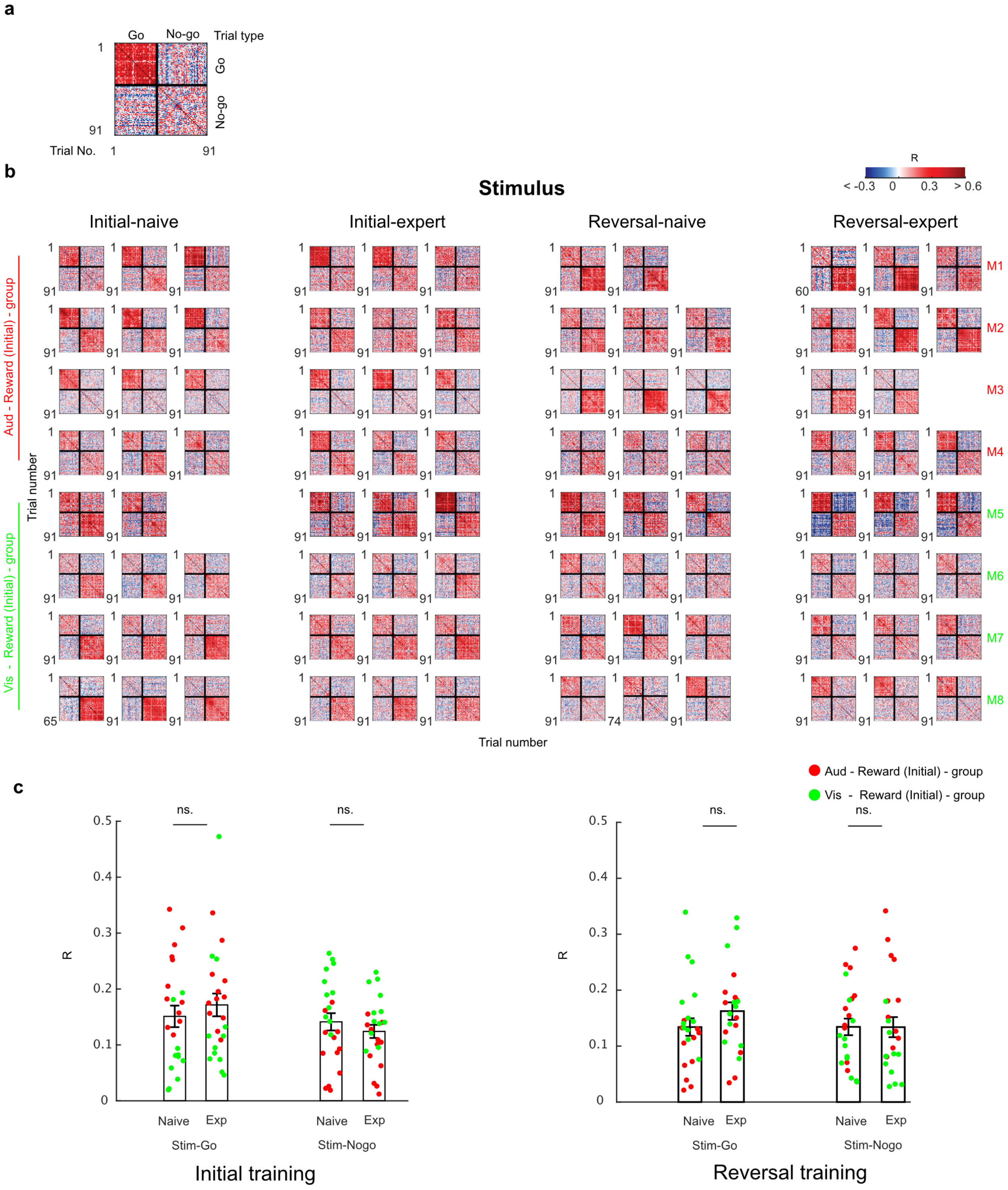

Supplementary Figure 7 | Stable evolution of MGB activity patterns during the stimulus period in the Go/Nogo learning task. (**a**) Representative trial-by-trial correlation matrix within and between Go/Nogo trials in one session. (**b**) Stimulus activity correlation matrices from all mice in different learning stages (Naive/Expert/Reversal-naive/Reversal-expert). Each matrix represents pair-wise population vector correlation (PVC) for all trials in one learning session. (**c**) Mean correlation value (R) in each session shown in (b) was grouped into initial (left) and reversal training (right). PVC in stimulus period in Expert and Reversal-expert did not significantly differ from Naive or Reversal-naive conditions, respectively (details in Supplementary Table 1). Visualization of counterbalanced stimulus-reward pairing. Both groups showed a consistent trend of activity patterns. Red: Mice trained on auditory-reward pairing in initial training session. Green: Mice trained on visual-reward pairing in initial training session.

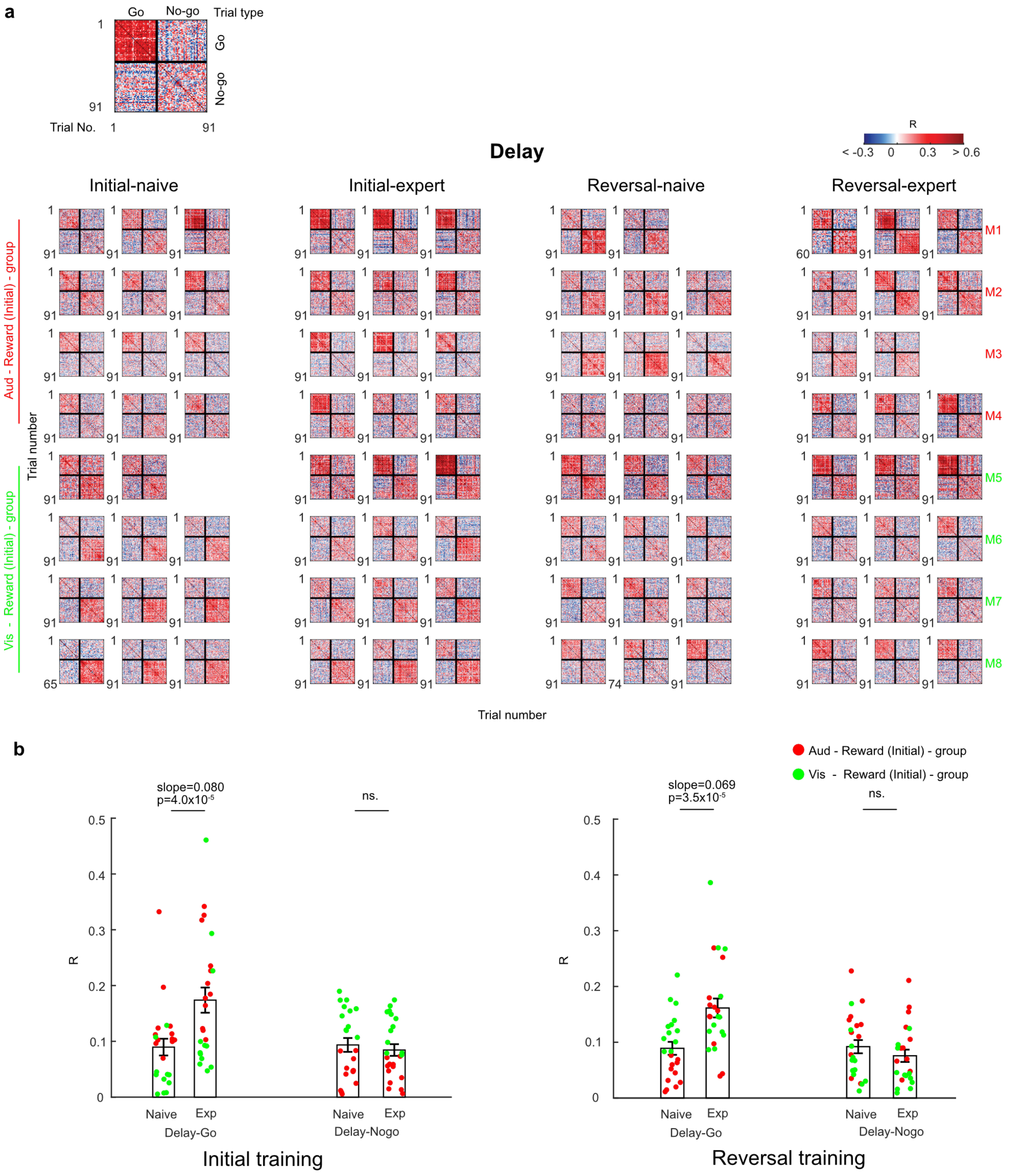

Supplementary Figure 8 | Distinct evolution of MGB activity patterns during the delay period in the Go/Nogo learning task. (**a**) Delay activity correlation matrices from all mice in different learning stages (Naive/Expert/Reversal-naive/Reversal-expert). Each matrix represents pair-wise population vector correlation (PVC) for all trials in one learning session. (**b**) Mean correlation value (R) in each session shown in (a) was grouped into initial (left) and reversal training (right). PVC in delay period in Go trial in Expert or Reversal-expert condition is higher than Naive or Reversal-naive respectively (slope > 0, $p < 0.01$, N = 8, details in Supplementary Table 1). Visualization of counterbalanced stimulus-reward pairing. Both groups showed consistent trend of activity patterns. Red dots: Mice trained on auditory-reward pairing in initial training session. Green dots: Mice trained on visual-reward pairing in initial training session.

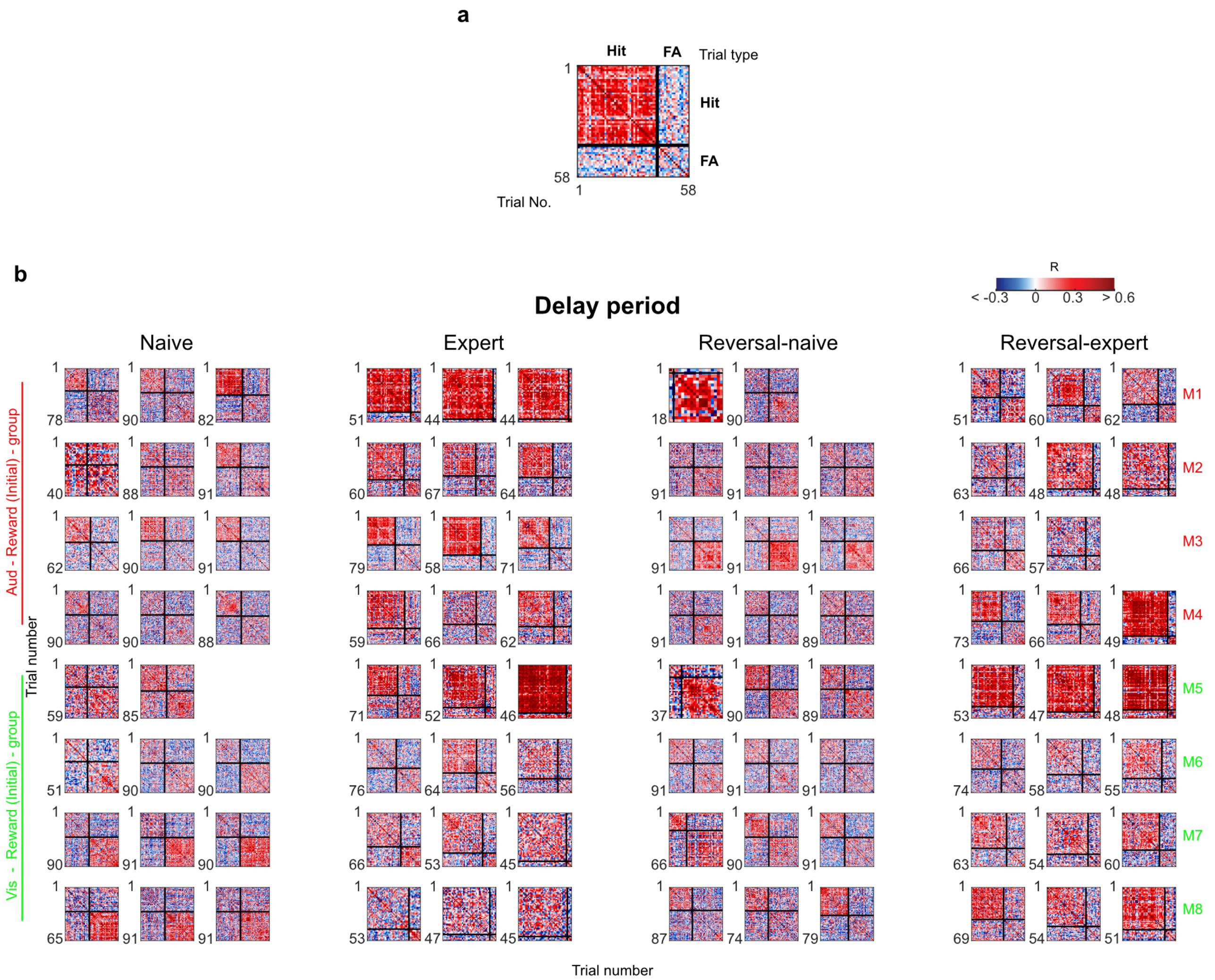

Supplementary Figure 9 | Coherence of MGB population activity is increased during the delay period of Hit but not False Alarm (FA) trials. (**a**) Representative trial-by-trial PVC matrix within and between Hit and False Alarm trials of one session. (**b**) Delay activity PVC matrices from all mice in different learning stages (Naive/Expert/Reversal-naive/Reversal-expert). Each matrix represent pair-wise PVC for all trials in one learning session. Summary data were shown in Fig.3 e, f and Supplementary Table 1.

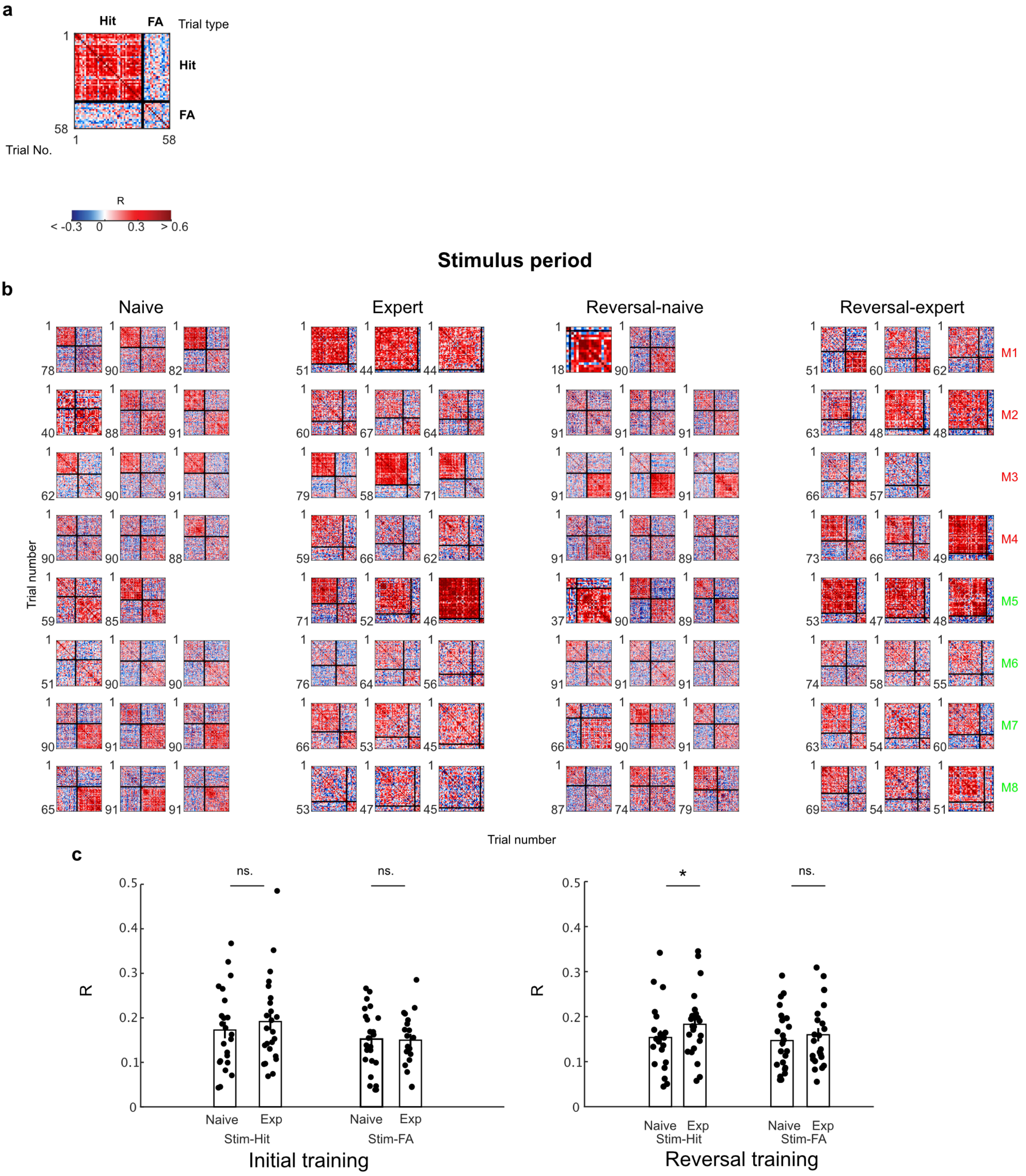

Supplementary Figure 10 | Coherence of MGB population activity is not increased during the stimulus period of Hit and False Alarm (FA) trials, but remodeled after reversal learning. (**a**) Representative trial-by-trial PVC matrix within and between Hit and False Alarm trials of one session. (**b**) Stimulus PVC matrices from all mice in different learning stages (Naive/Expert/Reversal-naive/Reversal-expert). Each matrix represent pair-wise PVC for all trials in one learning session. (**c**) Summary data. (N = 8, details in Supplementary Table 1).

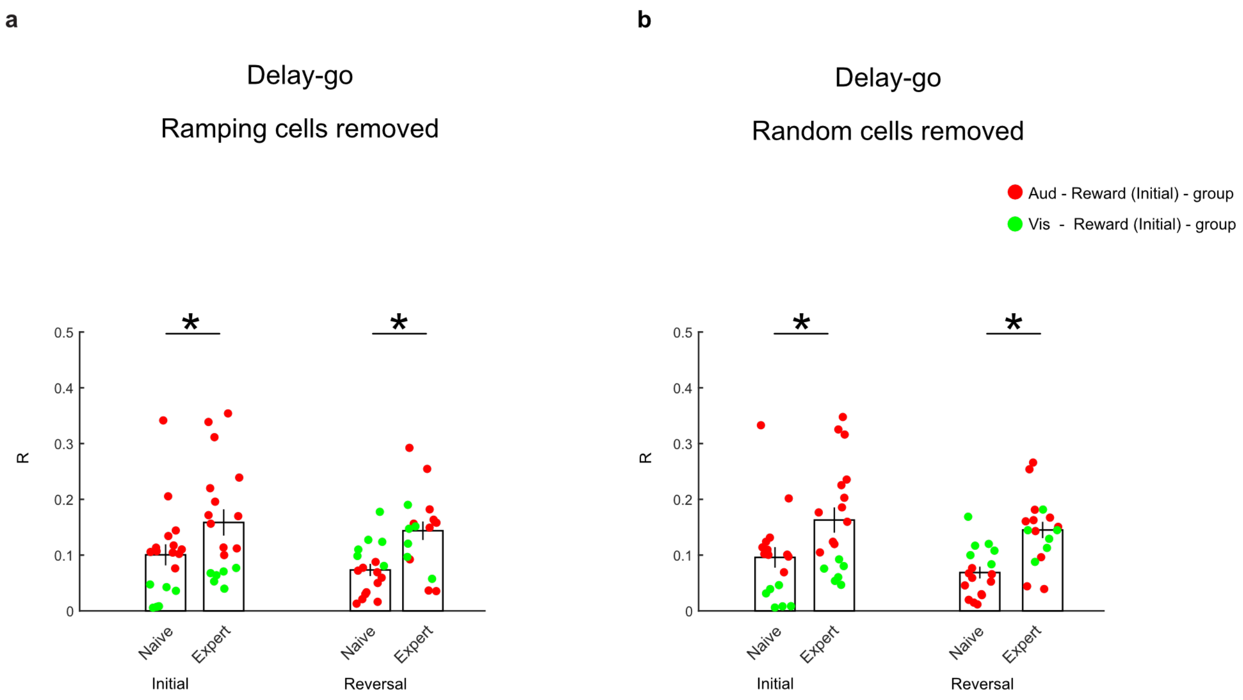

Supplementary Figure 11 | Increase of population vector correlation (PVC) in MGB during learning depends on MGB population activity and is not driven by ramping-cells. (**a**) Mean trial-by-trial PVC (R) during the delay period of Go trials in different learning stages. Increase of PVC persisted even if ramping cells (ramp-up and ramp-down, Fig. 2a, b) were removed (* $p < 0.01$, details in Supplementary Table 1). (**b**) Increase of PVC was dependent on the MGB population activity instead of subsets of neurons. Same number of cell as ramping cells (ramp-up + ramp-down) in each mouse were randomly removed before calculating PVC (number of random shuffles =30, * $p < 0.01$, details in Supplementary Table 1).

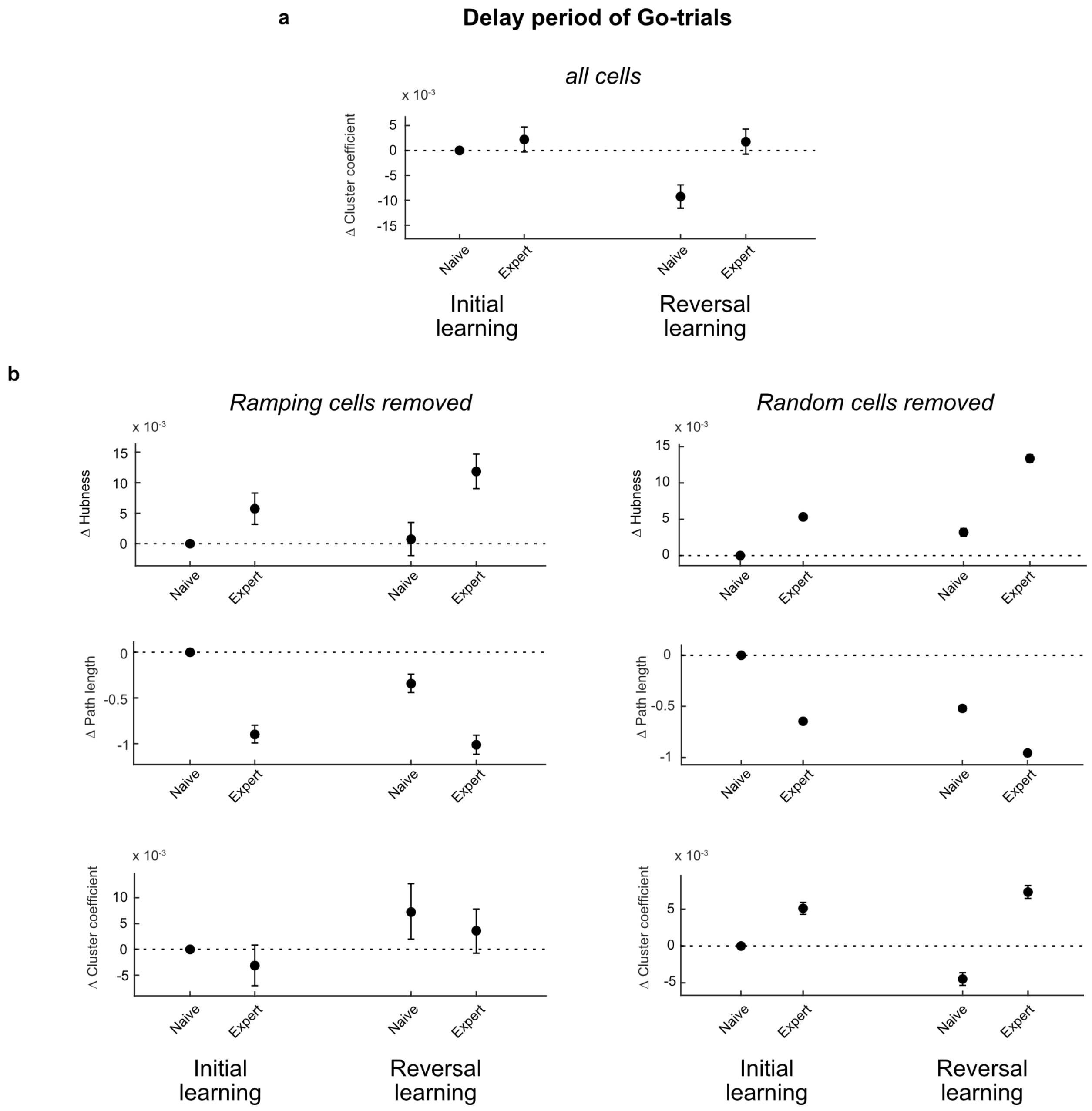

Supplementary Figure 12 | MGB co-activity network structure does not depend on ramping cells. (**a**) Decrease of local connectivity was observed specifically in reversal-naive learning stage when reward contingency was changed, suggesting a re-organization of pre-configured connectivity that was established in Initial-expert stage. (**b**) Ramp-cells were not the sole contributors of connectivity re-organization. Removing ramp-cells (ramp-up and ramp-down, Fig. 2a, b) or random cells did not abolish the change of network architecture which was an indication of MGB general ensemble remodeling during reward-association learning task (details in Methods).

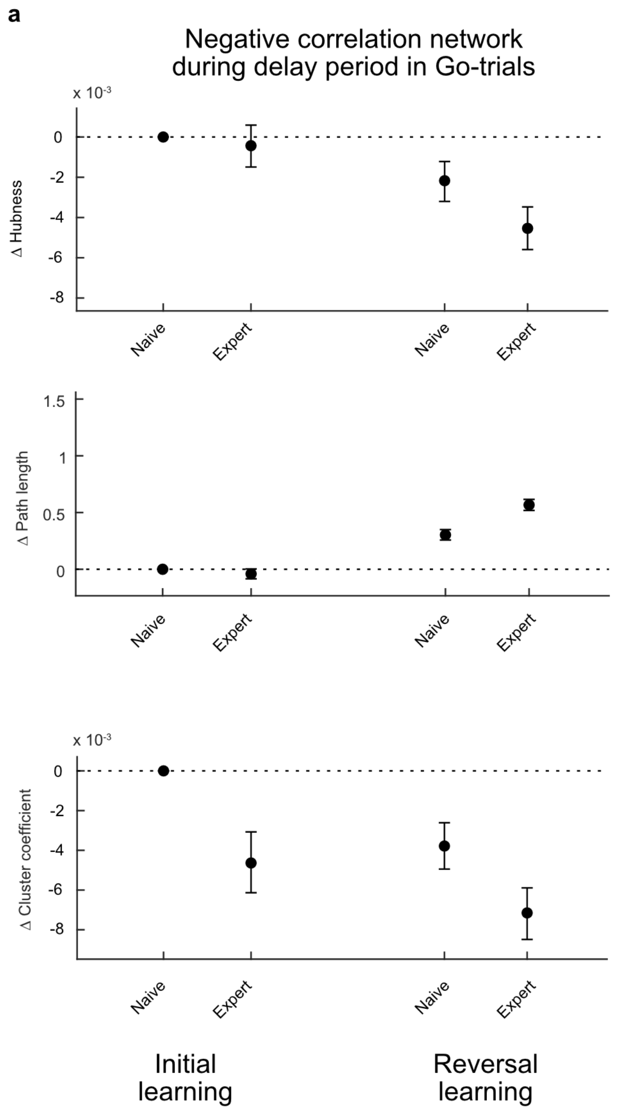

Supplementary Figure 13 | Negative correlation co-activity network during delay period in Go-trials. (**a**) There was no prominent change of global negative co-activity structure from Naive to Expert stage in Initial and Reversal learning phase which was in accordance to the increase of global positive co-activity network (detail in Methods). Local negative co-activity network became less connected from Naive to Expert stage suggesting an increase of connectivity during delay period when the mouse learned the task structure. Error bars: 95 % confidence interval of mean.

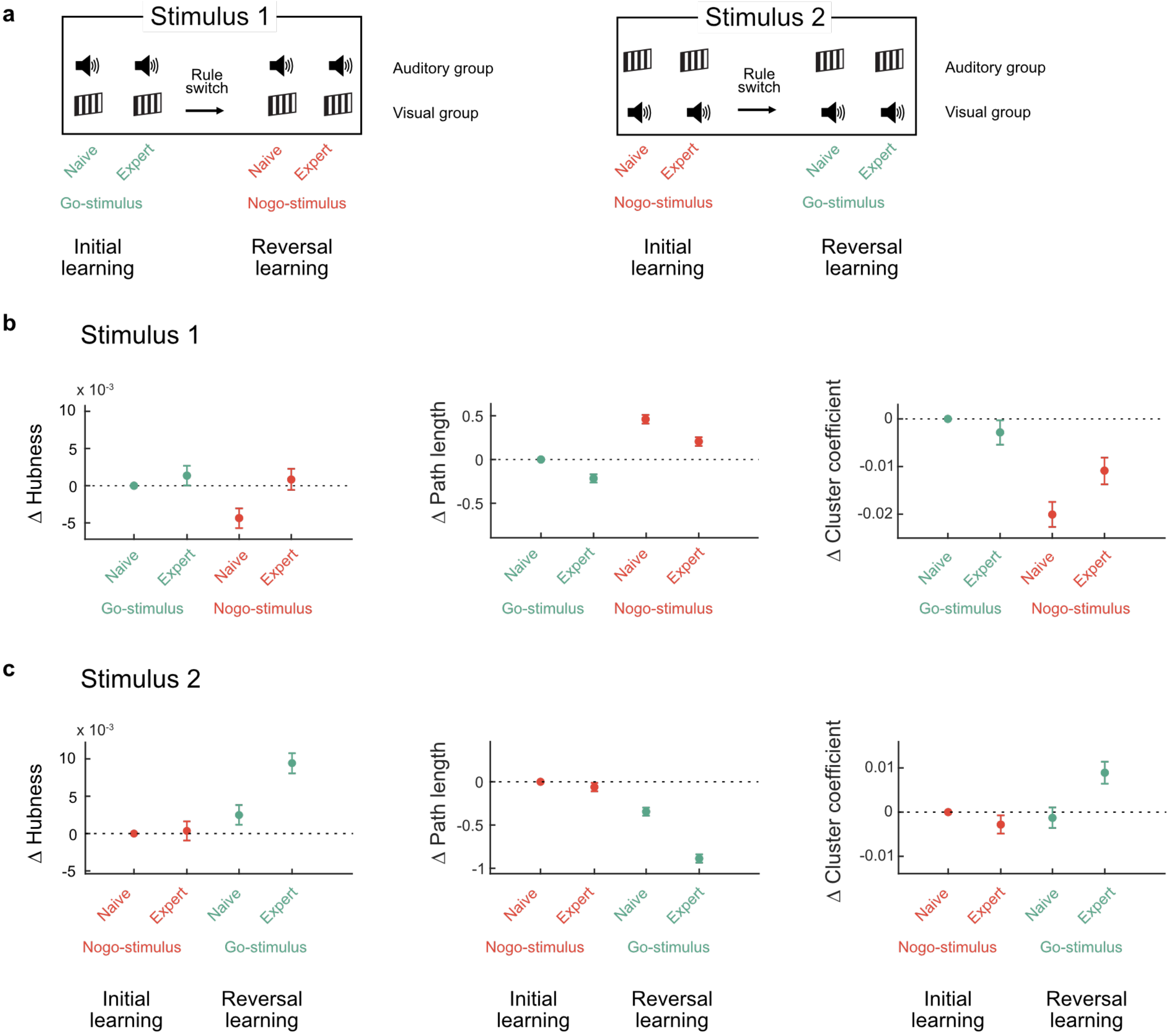

Supplementary Figure 14 | MGB co-activity networks during the stimulus period. (**a**) Experimental design. (**b** and **c**) Change of hubness, path length and cluster coefficient across all neuron pairs in relation to the naive Go (or) Nogo condition (detail in Materials and Methods). There was no obvious change of global and local connectivity in initial training phase during stimulus presentation period for within group identical stimulus 1 and 2. Conversely in reversal learning, global and local connectivity of both Go and Nogo stimuli were strengthened. Neural representation of stimuli that were associated or dissociated with reward contingency were remodeled in the reversal naive phase. Error bars: 95 % confidence interval of mean.

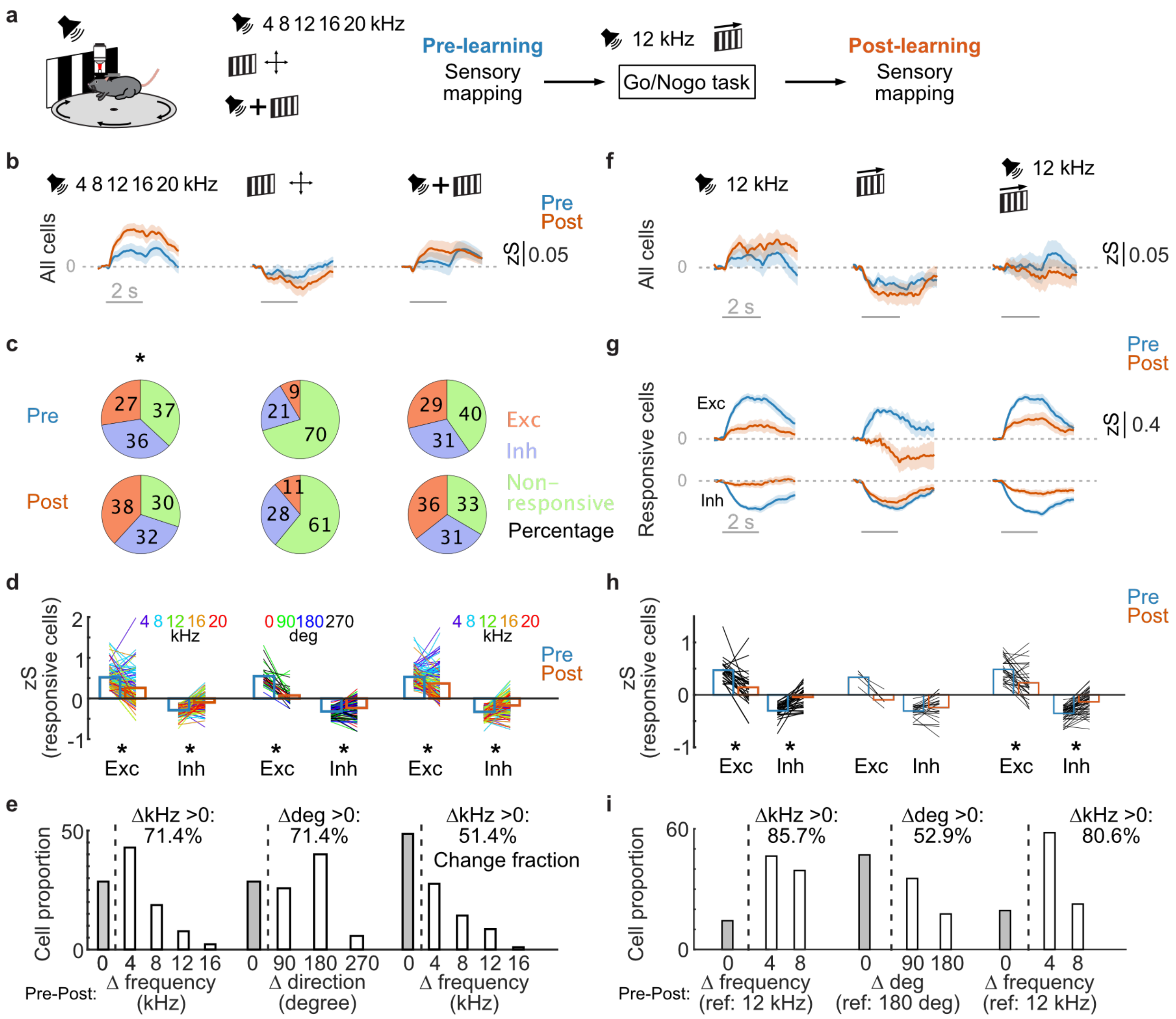

Supplementary Figure 15 | Sensory coding in MGB is not biased to the reward-associated stimuli in non-rewarded sensory mapping sessions. (**a**) Schematics of sensory mapping. Various auditory and visual stimuli were presented as unisensory stimulus, and the combination of them were presented as multisensory stimulus. (**b**) Average population calcium activity in uni- and multi-sensory trials before and after learning (n = 233 cells, 6 mice). (**c**) Proportions of the excited and inhibited cells to auditory, visual and multi-sensory stimuli. (**d**) Transition of the sensory response amplitude from the cells showing the significant response at pre-learning stage were plotted. Each line represents sensory responses before and after learning from the same cell for a specific stimulus feature (tone frequency or grating direction). (**e**) Changes of the best frequency/direction tuning across learning. (**f**) Averaged population calcium activity to the reward-conditioned sensory stimuli (12 kHz tone, rightward grating, combination of them, n = 233 cells, 6 mice). (**g**) Average population responses of the excited or inhibited cells to the conditioned stimuli. (**h**) Transition of the sensory response amplitude to the conditioned stimuli. Each line represents the sensory response of the same cell before and after learning. (**i**) Changes of the best frequency/direction tuning referred to the conditioned stimuli. Statistical details in Supplementary Table 1.

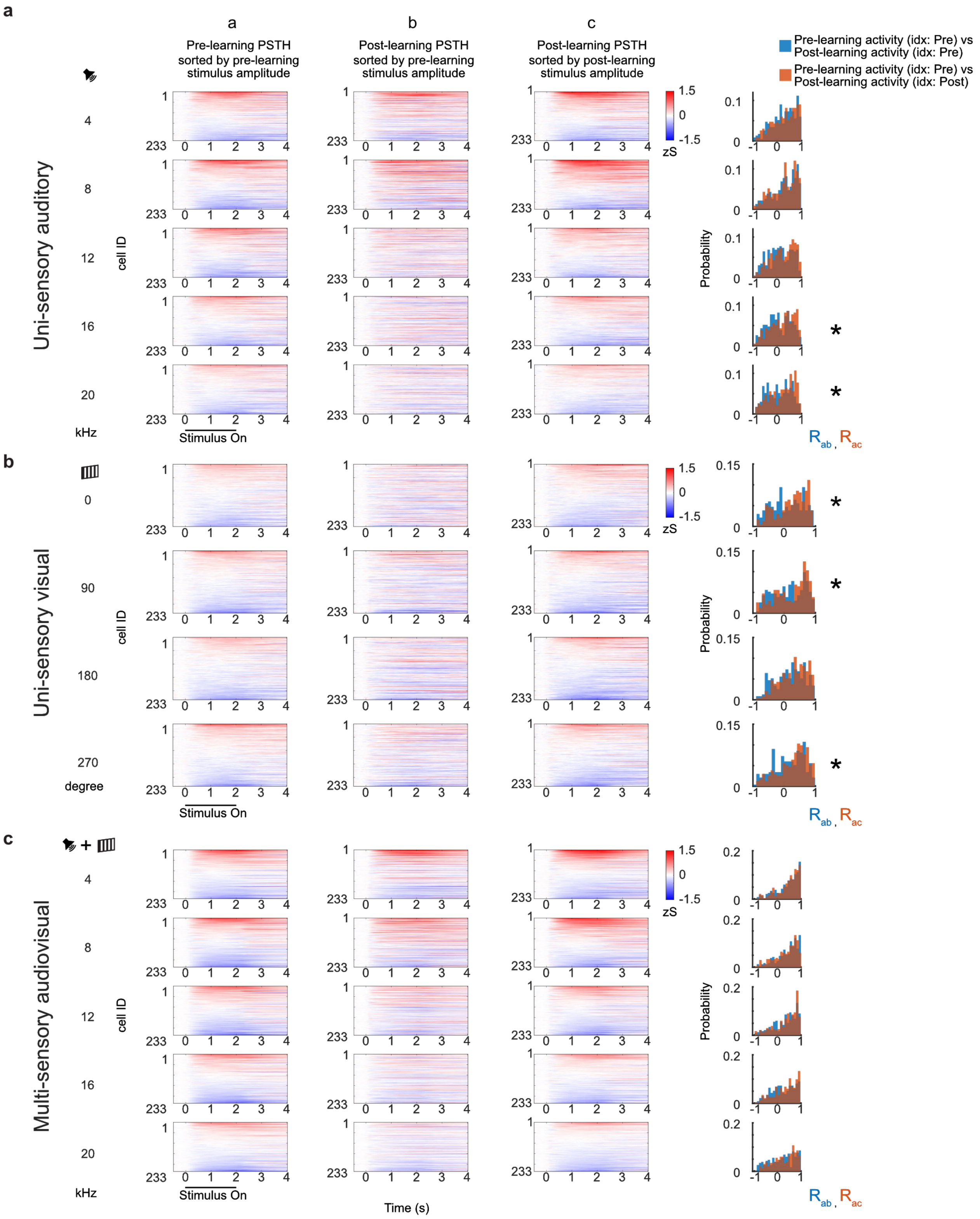

Supplementary Figure 16 | MGB single cell responses across sensory mapping. (**a**) Heat-map shows average peri-stimulus time histogram (PSTH) of cell response in pre-learning for uni-sensory auditory stimulus sorted by pre-learning response amplitude during stimulus presentation (column a). Same sorting index was used to sort post-learning PSTH (column b). Reduced response amplitude with same sorting index indicated a remapping of sensory response to the same auditory frequency. Post-learning responses were not diminished after learning if cells were sorted with amplitude in post-learning session (column c). Correlation of cell response time series using two different indexes was compared to quantify the extent of remapping (R_{ab}:R_{ac}, 2-sample ks-test). (**b, c**) Same measurement for uni-sensory visual response and multi-sensory response.

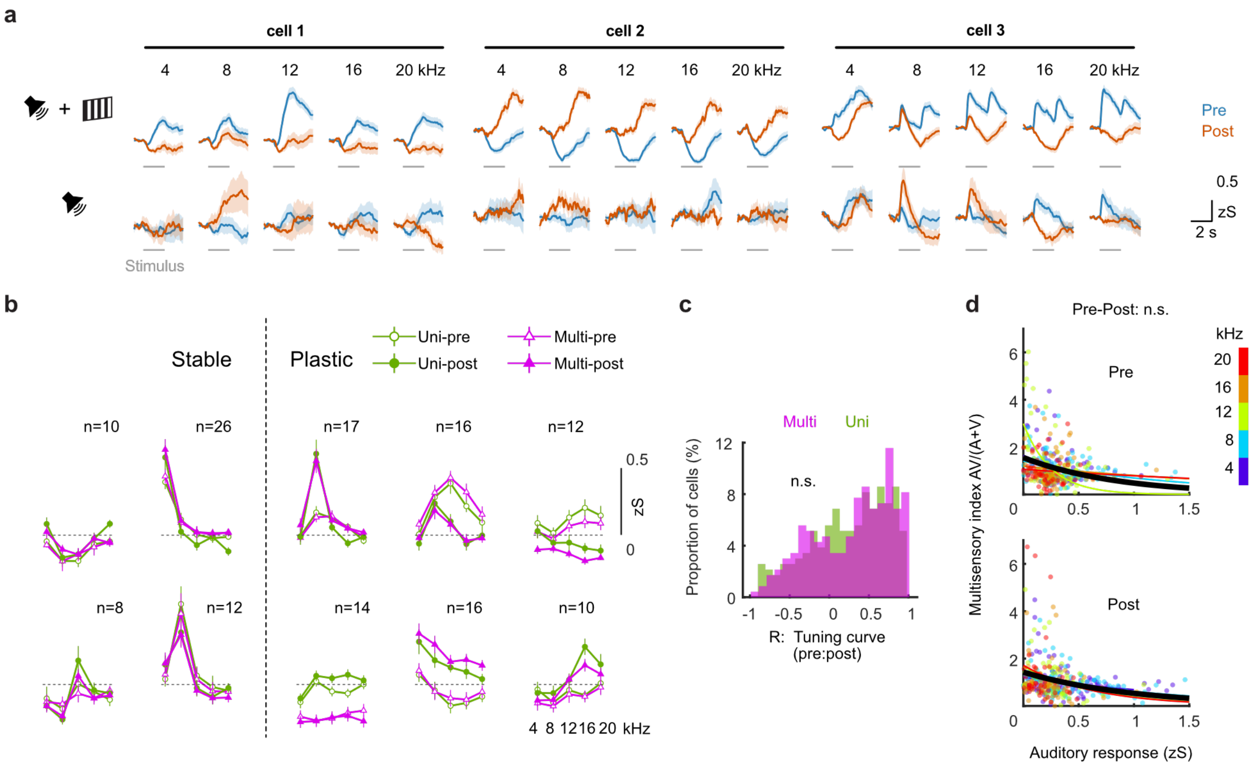

Supplementary Figure 17 | Single cell tuning properties are not biased to the reward-associated stimuli in non-rewarded sensory mapping sessions. (**a**) Example sensory responses of individual MGB neurons to auditory and multisensory stimuli. Individual neurons showed stable or plastic sensory response patterns to auditory and multisensory stimuli. (**b**) Transition of tone-frequency tuning across learning. The neurons showing a similar changing pattern of frequency tuning were grouped by k-means clustering. (**c**) Distribution of tuning curve similarity of individual neurons across learning. Tuning curve similarities are comparable in auditory and multisensory trials. R is Pearson's correlation coefficient between pre- and post-learning tuning curve (n = 233 cells, 6 mice). (**d**) Multisensory index as a function of unisensory tone response. Two-dimension distribution of unisensory response (auditory) versus multisensory index was compared between pre-learning and post-learning conditions (5 frequencies were pooled, $p > 0.05$, details in Supplementary Table 1).

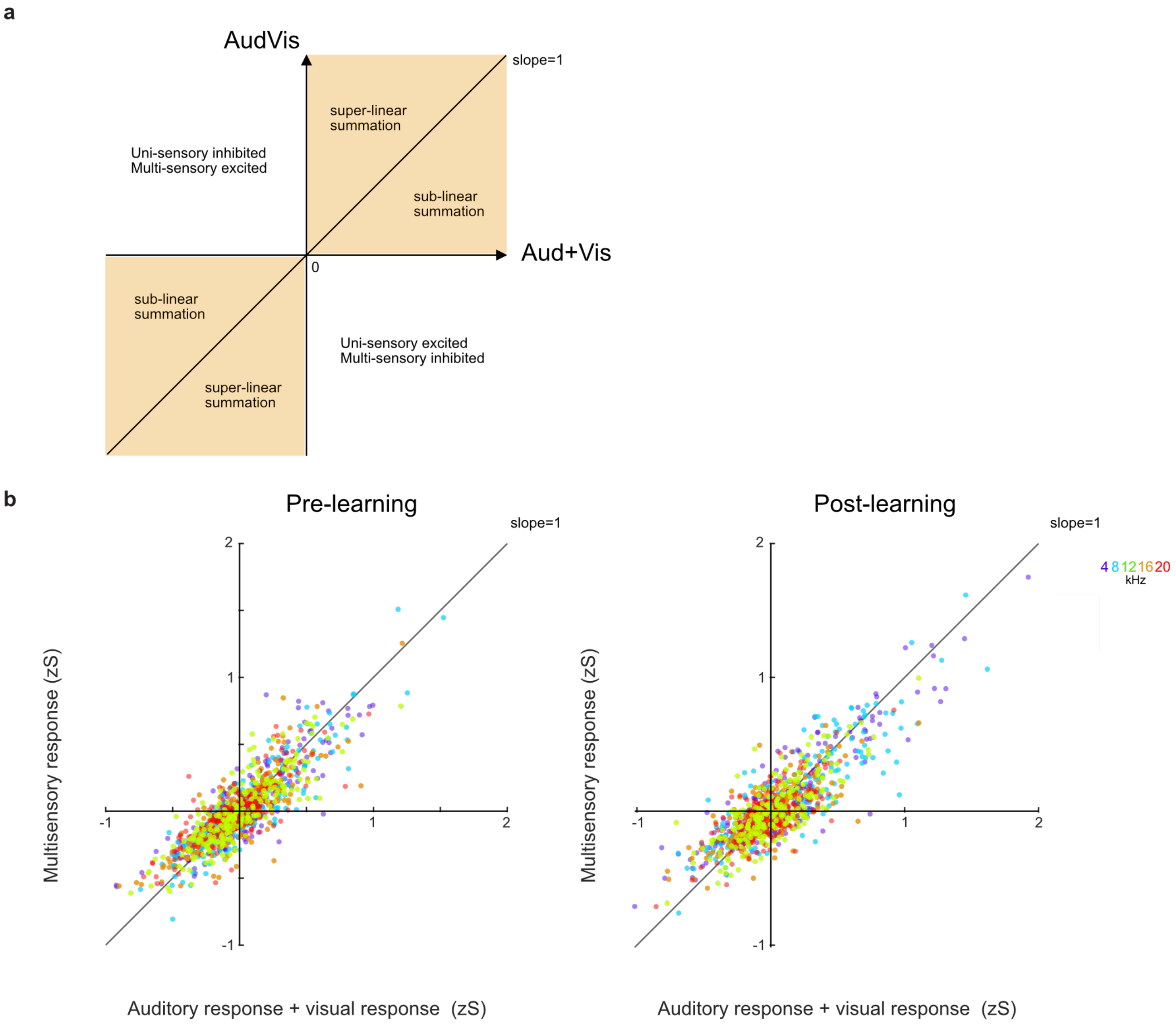

Supplementary Figure 18 | Multisensory integration remains unchanged after cross-modal reward learning. (**a**) Multi-sensory integration principle is described in each quadrant. Data in 1st and 3rd quadrant were included in analysis in Supp;ementary Figure 17d. (**b**) Average response of Multi-sensory trials (Audiovisual trials) for each cell and for each auditory frequency were plotted against the sum of average response of Uni-sensory trials (Auditory and Visual trials). Diagonal line represents unity between multisensory responses and the linear sum of individual unisensory responses. Response distribution was not different between pre-learning and post-learning sessions (2-dimensional 2-sample KS-test, details in Supplementary Table 1). Each dot indicates a cell (repeat measurement form 1-3 sessions).

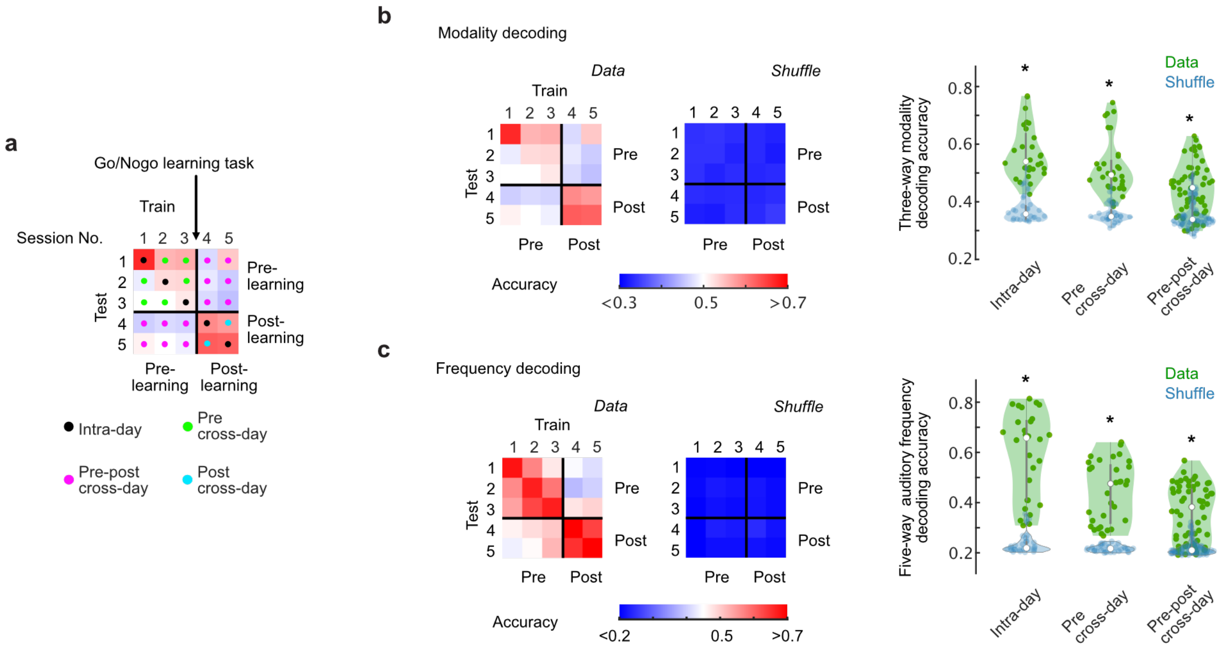

Supplementary Figure 19 | The total MGB population reliably encodes sensory information. (**a**) Decoding design matrix. Linear-SVM decoder was trained and tested in a session-by-session manner. (**b**) Three-way modality decoding. Left: Matrix of modality decoding accuracy from one representative mouse. Middle: Matrix of modality decoding accuracy from the same mouse generated by the shuffled data. Right: Decoding accuracies grouped by intra-day, pre cross-day and pre-post cross-day conditions. Modality decoding accuracies were above chance (*$p < 0.01$). Each dot represents the one session data from one mouse. Shuffle data showed the chance level decoding accuracy. (**c**) Five-way tone-frequency decoding. The panel structure is the same as (b). Frequency decoding accuracies were above chance (*$p < 0.01$, details in Supplementary Table 1).

Supplementary Tables

Table. S1. Summary of statistical tests

Fig	Method	Statistics value	df	p	bonferroni-corrected p	detail description	notes
Fig.1f	Signed-rank test	2.00E+01		8.44E-01	NA	N of session to reach criterion between initial and reversal learning	
Fig.1l, left	Chi-square test	2.95E+01		1.74E-06	3.48E-06	Initial learning, cell type ratio	2x bonferroni correction
Fig.1l, Right	Chi-square test	2.66E+01		7.22E-06	1.44E-05	Reversal learning, cell type ratio	2x bonferroni correction
Fig.1m, Left	Signed-rank test	0.00E+00		7.80E-03	1.56E-02	Initial learning, Go cell ratio	2x bonferroni correction
Fig.1m, Right	Signed-rank test	0.00E+00		7.80E-03	1.56E-02	Reversal learning, Go cell ratio	2x bonferroni correction
Fig3. a-d	Linear Mix Model (fix:Nai-Exp, random: Mouse ID)	1.62E-02	45	3.18E-01	1.27E+00	Main-effect slope, Go,Stim	4x bonferroni correction
		8.03E-02	45	9.89E-06	3.96E-05	Main-effect slope, Go,Delay	4x bonferroni correction
	Linear Mix Model (fix:RevNai-RevExp, random: Mouse ID)	2.55E-02	44	2.53E-02	1.01E-01	Main-effect slope, Go,Stim	4x bonferroni correction
		6.88E-02	44	8.79E-06	3.52E-05	Main-effect slope, Go,Delay	4x bonferroni correction
Fig3. e,f	Linear Mix Model (fix:Nai-Exp, random: Mouse ID)	7.51E-02	45	3.49E-05	1.40E-04	Main-effect slope, Hit,Delay	4x bonferroni correction
		1.27E-02	40	9.72E-02	3.89E-01	Main-effect slope, FA, Delay	4x bonferroni correction
	Linear Mix Model (fix:RevNai-RevExp, random: Mouse ID)	6.36E-02	43	5.43E-05	2.17E-04	Main-effect slope, Hit,Delay	4x bonferroni correction
		1.47E-02	44	2.47E-01	9.88E-01	Main-effect slope, FA, Delay	4x bonferroni correction
Fig3. g	Wilcoxon Rank Sum Test	9.10E+01		3.08E-01	6.15E-01	Expert-Hit, Lick/Nolick	2x bonferroni correction
		9.50E+01		4.73E-01	9.45E-01	Naive-Hit, Lick/Nolick	PVC-Hit-Lick/Nolick
Fig3. h	Linear Mix Model (fix:Nai-Exp, random: Mouse ID)	5.81E-02	34	1.07E-03	2.14E-03	Main-effect slope, Go,Delay (Nai Exp)	2x bonferroni correction (removing ramp cells)
		6.60E-02	32	2.47E-04	4.95E-04	Main-effect slope, Go,Delay (RevNai RevExp)	

Fig	Method	Statistics value	df	p	bonferroni-corrected p	detail description	notes
fig. S1c	Signed-rank test	3.50E+01		1.56E-02	3.13E-02	Hit RT between naive and expert phase	2x bonferroni correction
fig. S1f	Signed-rank test	3.50E+01		1.56E-02	3.13E-02	Hit RT between naive and expert phase	2x bonferroni correction
		F (DFn, DFd)	DF	P value			
fig.S2f	2-way ANOVA	F (9, 81) = 6.283	9	P<0.0001	NA	Time x Group	
		F (3.559, 32.03) = 29.37	9	P<0.0001	NA	Time	

		F (1, 9) = 20.23	1 P=0.0015	NA	Group	
fig. S3c	Chi-square	1.84E+01	3.57E-04	7.14E-04	Proportion of stimulus-responsive cell in Aud-reward 1st in Initial learning	2x bonferroni correction
fig. S3c	Chi-square	2.85E+01	2.80E-06	5.60E-06	Proportion of stimulus-responsive cell in Aud-reward 1st in Reversal learning	2x bonferroni correction
fig. S3d	Chi-square	1.75E+01	5.57E-04	1.11E-03	Proportion of stimulus-responsive cell in Vis-reward 1st in Initial learning	2x bonferroni correction
fig. S3d	Chi-square	1.20E+01	7.50E-03	1.50E-02	Proportion of stimulus-responsive cell in Vis-reward 1st in Reversal learning	2x bonferroni correction
fig. S6c, left	ks-test (2 sample)	1.24E-01	7.36E-02	2.21E-01	r value distributions between naive and expert phase during the stimulus period in Hit trials, locmotion	3x bonferroni correction
fig. S6c, left	ks-test (2 sample)	1.81E-01	1.73E-03	5.19E-03	r value distributions between naïve and expert phase during the delay period in Hit trials, locomotion	3x bonferroni correction
fig. S6c, left	ks-test (2 sample)	6.19E-02	8.04E-01	2.41E+00	r value distributions between naïve and expert phase during the ITI period in Hit trials, locomotion	3x bonferroni correction
fig. S6c, right	ks-test (2 sample)	1.33E-01	4.35E-02	1.30E-01	r value distributions between naïve and expert phase during the stimulus period in Hit trials, locmotion	3x bonferroni correction
fig. S6c, right	ks-test (2 sample)	1.33E-01	4.35E-02	1.30E-01	r value distributions between naïve and expert phase during the delay period in Hit trials, locomotion	3x bonferroni correction
fig. S6c, right	ks-test (2 sample)	7.14E-02	6.42E-01	1.93E+00	r value distributions between naïve and expert phase during the ITI period in Hit trials, locomotion	3x bonferroni correction
fig. S6d, left	ks-test (2 sample)	1.05E-01	1.88E-01	5.64E-01	r value distributions between naïve and expert phase during the stimulus period in Hit trials	3x bonferroni correction
fig. S6d, left	ks-test (2 sample)	1.57E-01	9.80E-03	2.94E-02	r value distributions between naïve and expert phase during the delay period in Hit trials	3x bonferroni correction
fig. S6d, left	ks-test (2 sample)	1.00E-01	2.32E-01	6.95E-01	r value distributions between naïve and expert phase during the ITI period in Hit trials	3x bonferroni correction
fig. S6d, right	ks-test (2 sample)	1.81E-01	1.73E-03	5.19E-03	r value distributions between naïve and expert phase during the stimulus period in Hit trials	3x bonferroni correction
fig. S6d, right	ks-test (2 sample)	8.10E-02	4.80E-01	1.44E+00	r value distributions between naïve and expert phase during the delay period in Hit trials	3x bonferroni correction
fig. S6d, right	ks-test (2 sample)	1.00E-01	2.32E-01	6.95E-01	r value distributions between naïve and expert phase during the ITI period in Hit trials	3x bonferroni correction

fig. S6e, left	signed-rank	8.49E+03		3.32E-03	9.96E-03	Delta of absolute r value during the stimulus period	3x bonferroni correction
fig. S6e, left	signed-rank	5.96E+03		1.00E-08	3.00E-08	Delta of absolute r value during the delay period	3x bonferroni correction
fig. S6e, left	signed-rank	1.08E+04		7.46E-01	2.24E+00	Delta of absolute r value during the ITI period	3x bonferroni correction
fig. S6e, right	signed-rank	8.50E+03		3.47E-03	1.04E-02	Delta of absolute r value during the stimulus period	3x bonferroni correction
fig. S6e, right	signed-rank	7.69E+03		1.24E-04	3.72E-04	Delta of absolute r value during the delay period	3x bonferroni correction
fig. S6e, right	signed-rank	9.31E+03		4.44E-02	1.33E-01	Delta of absolute r value during the ITI period	3x bonferroni correction
fig. S6f, left	signed-rank	1.12E+04		9.18E-01	2.75E+00	Delta of absolute r value during the stimulus period	3x bonferroni correction
fig. S6f, left	signed-rank	9.74E+03		1.29E-01	3.86E-01	Delta of absolute r value during the delay period	3x bonferroni correction
fig. S6f, left	signed-rank	9.78E+03		1.42E-01	4.25E-01	Delta of absolute r value during the ITI period	3x bonferroni correction
fig. S6f, right	signed-rank	1.30E+04		3.22E-02	9.66E-02	Delta of absolute r value during the stimulus period	3x bonferroni correction
fig. S6f, right	signed-rank	1.08E+04		7.71E-01	2.31E+00	Delta of absolute r value during the delay period	3x bonferroni correction
fig. S6f, right	signed-rank	8.16E+03		9.40E-04	2.82E-03	Delta of absolute r value during the ITI period	3x bonferroni correction
fig. S6g, left	Chi-square	5.56E+01		9.09E-14	1.82E-13	Frequency of lick and non-lick trial between naive and expert in initial learning	2x bonferroni correction
fig. S6g, right	Chi-square	4.34E+01		4.36E-11	8.72E-11	Frequency of lick and non-lick trial between naive and expert in reversal learning	2x bonferroni correction
fig. S7	Linear Mix Model (fix:Nai-Exp, random: Mouse ID)	1.62E-02	45	3.18E-01	1.27E+00	Main-effect slope, Go,Stim	4x bonferroni correction
		-1.96E-02	45	4.98E-02	1.99E-01	Main-effect slope, NoGo, Stim	
	Linear Mix Model (fix:RevNai-RevExp, random: Mouse ID)	2.55E-02	44	2.53E-02	1.01E-01	Main-effect slope, Go,Stim	
		-4.29E-03	44	6.87E-01	2.75E+00	Main-effect slope, NoGo, Stim	
fig. S8	Linear Mix Model (fix:Nai-Exp, random: Mouse ID)	8.03E-02	45	9.89E-06	3.96E-05	Main-effect slope, Go,Delay	4x bonferroni correction
		-1.10E-02	45	1.07E-01	4.28E-01	Main-effect slope, NoGo, Delay	
	Linear Mix Model (fix:RevNai-RevExp, random: Mouse ID)	6.88E-02	44	8.79E-06	3.52E-05	Main-effect slope, Go,Delay	
		-1.85E-02	44	2.21E-02	8.85E-02	Main-effect slope, NoGo, Delay	
fig. S10d	Linear Mix Model (fix:Nai-Exp, random: Mouse ID)	1.52E-02	45	3.50E-01	1.40E+00	Main-effect slope, Hit,Stim	4x bonferroni correction
		7.73E-04	40	9.35E-01	3.74E+00	Main-effect slope, FA, Stim	
	Linear Mix Model (fix:RevNai-RevExp, random: Mouse ID)	2.86E-02	43	9.31E-03	3.73E-02	Main-effect slope, Hit,Stim	
		9.81E-03	44	3.61E-01	1.45E+00	Main-effect slope, FA, Stim	
fig. S11a	Linear Mix Model (fix:Nai-Exp, random: Mouse ID)	5.81E-02	34	1.07E-03	2.14E-03	Main-effect slope, Go,Delay (Nai Exp)	2x bonferroni correction (removing ramp cells)
		6.60E-02	32	2.47E-04	4.95E-04	Main-effect slope, Go,Delay (RevNai RevExp)	

fig. S11b	Linear Mix Model (fix:Nai-Exp, random: Mouse ID)	6.70E-02	34	1.69E-04	3.37E-04	Main-effect slope, Go,Delay (Nai Exp), random removal	2x bonferroni correction (randomly removing ramp cells)
		7.24E-02	32	2.27E-05	4.55E-05	Main-effect slope, Go,Delay (RevNai RevExp), random removal	
fig. S15c	Chi-square Test	6.24E+00		4.41E-02	NA	Uni-Aud, Pre:Post learning	Response cell fraction
		4.61E+00		9.98E-02	NA	Uni-Vis, Pre:Post learning	
		3.20E+00		2.02E-01	NA	Multi-AudVis, Pre:Post learning	
fig. S15d	Rank sum test	1.38E+04		7.62E-10	1.52E-09	Uni-Aud-Exc, Pre:Post learning	2x bonferroni correction
		1.50E+04		4.95E-20	9.90E-20	Uni-Aud-Inh, Pre:Post learning	2x bonferroni correction
		1.13E+03		7.32E-08	1.46E-07	Uni-Vis-Exc, Pre:Post learning	2x bonferroni correction
		6.51E+03		9.20E-04	1.84E-03	Uni-Vis-Inh, Pre:Post learning	2x bonferroni correction
		1.87E+04		2.55E-06	5.10E-06	Multi-AudVis-Exc, Pre:Post learning	2x bonferroni correction
		3.03E+04		1.44E-19	2.88E-19	Multi-AudVis-Inh, Pre:Post learning	2x bonferroni correction
fig. S15h	Rank sum test	9.31E+02		9.88E-06	1.98E-05	Uni-12kHz-Exc, Pre:Post learning	2x bonferroni correction
		7.15E+02		5.69E-07	1.14E-06	Uni-12kHz-Inh, Pre:Post learning	2x bonferroni correction
		NA		NA	NA	Uni-180deg-Exc, Pre:Post learning	n=4, sample too small for ranksum test
		4.66E+02		1.04E-01	2.08E-01	Uni-180deg-Inh, Pre:Post learning	2x bonferroni correction
		1.15E+03		4.98E-04	9.96E-04	Multi-12kHz180deg-Exc, Pre:Post learning	2x bonferroni correction
		1.65E+03		8.01E-07	1.60E-06	Multi-12kHz180deg-Inh, Pre:Post learning	2x bonferroni correction
fig. S16	ks test (2-sample)	5.58E-02		8.51E-01	4.26E+00	D value, Uni-Aud: 4 kHz	R-Pre-learning activity (idx: Pre) vs R-Post-learning activity (idx: Pre)
		4.29E-02		9.80E-01	4.90E+00	D value, Uni-Aud: 8 kHz	
		1.42E-01		1.67E-02	8.34E-02	D value, Uni-Aud: 12 kHz	
		1.63E-01		3.50E-03	1.75E-02	D value, Uni-Aud: 16 kHz	
		1.55E-01		6.71E-03	3.36E-02	D value, Uni-Aud: 20 kHz	
		2.10E-01		5.21E-05	2.09E-04	D value, Uni-Vis: 0 deg	R-Pre-learning activity (idx: Pre) vs R-Post-learning activity (idx: Pre)
		1.63E-01		3.50E-03	1.40E-02	D value, Uni-Vis: 90 deg	
		1.37E-01		2.22E-02	8.87E-02	D value, Uni-Vis: 180 deg	
		1.63E-01		3.50E-03	1.40E-02	D value, Uni-Vis: 270 deg	
		6.01E-02		7.82E-01	3.91E+00	D value, Multi-Aud: 4 kHz	R-Pre-learning activity (idx: Pre) vs R-Post-learning activity (idx: Pre)
		8.15E-02		4.06E-01	2.03E+00	D value, Multi-Aud: 8 kHz	
		8.58E-02		3.43E-01	1.71E+00	D value, Multi-Aud: 12 kHz	
		1.46E-01		1.24E-02	6.21E-02	D value, Multi-Aud: 16 kHz	
		1.16E-01		8.11E-02	4.06E-01	D value, Multi-Aud: 20 kHz	
fig. S17c	ks test (2-sample)	7.73E-02		4.75E-01		R-Multi-sensory vs R-Uni-sensory	all cells were included
		5.62E-02		1.04E-01		Multi-sensory response distribution (2d, x: Aud+Vis, y: index), pre vs post	all frequencies were mixed
fig. S17d	2-dimension K-S test (2-sample)	9.57E-02		1.85E-01		Multi-sensory index distribution (2d, x:Aud, y: index), pre vs post	all frequencies were mixed
fig. S18	2-dimension K-S test (2-sample)	5.62E-02		1.04E-01		Multi-sensory response distribution (2d, x: Aud+Vis, y: index), pre vs post	all frequencies were mixed

fig. S19b	Wilcoxon Rank Sum Test	1.18E+03	3.66E-10	1.10E-09	Intra-day, data:shuffle, modality decoding	3x bonferroni correction
		1.54E+03	2.18E-11	6.54E-11	Pre-cross day, data:shuffle, modality decoding	3x bonferroni correction
		5.00E+03	2.15E-08	6.45E-08	PrePostCrossDay, data:shuffle, modality decoding	3x bonferroni correction
fig. S19c	Wilcoxon Rank Sum Test	1.18E+03	3.29E-10	9.88E-10	Intra-day, data:shuffle, frequency decoding	3x bonferroni correction
		1.55E+03	6.51E-12	1.95E-11	Pre-cross day, data:shuffle, frequency decoding	3x bonferroni correction
		5.27E+03	3.19E-12	9.58E-12	PrePostCrossDay, data:shuffle, frequency decoding	3x bonferroni correction

All signed-rank and rank-sum tests are 2-side test
