# Supplementary material for: Network state changes in sensory thalamus represent learned outcomes
Source: Nat Commun. 2024 Sep 7;15:7830. doi: 10.1038/s41467-024-51868-8 (PMC11380690; doi:10.1038/s41467-024-51868-8)
Supplement: Supplementary file 3 — Description of additional supplementary files [file 41467_2024_51868_MOESM3_ESM.pdf]

<u>Description of Additional Supplementary Files</u>

Supplementary Movie 1: Two-photon calcium imaging in auditory thalamus.
GRIN-lens-based longitudinal two-photon calcium imaging of neuronal activity in medial geniculate body during a cross-modal reward associative learning paradigm. The same field of view is imaged in the naïve (day1) and expert (day 9) stage of the example mouse.
