# Supplementary material for: Network state changes in sensory thalamus represent learned outcomes
Source: Nat Commun. 2024 Sep 7;15:7830. doi: 10.1038/s41467-024-51868-8 (PMC11380690; doi:10.1038/s41467-024-51868-8)
Supplement: Supplementary file 5 — Reporting summary [file 41467_2024_51868_MOESM5_ESM.pdf]

nature portfolio

Corresponding author(s): Jan Gründemann

Last updated by author(s): Jul 23, 2024

Reporting Summary

Nature Portfolio wishes to improve the reproducibility of the work that we publish. This form provides structure for consistency and transparency in reporting. For further information on Nature Portfolio policies, see our Editorial Policies and the Editorial Policy Checklist.

Statistics

For all statistical analyses, confirm that the following items are present in the figure legend, table legend, main text, or Methods section.

n/a	Confirmed	
☐	☒	The exact sample size (*n*) for each experimental group/condition, given as a discrete number and unit of measurement
☐	☒	A statement on whether measurements were taken from distinct samples or whether the same sample was measured repeatedly
☐	☒	The statistical test(s) used AND whether they are one- or two-sided<br>*Only common tests should be described solely by name; describe more complex techniques in the Methods section.*
☐	☒	A description of all covariates tested
☐	☒	A description of any assumptions or corrections, such as tests of normality and adjustment for multiple comparisons
☐	☒	A full description of the statistical parameters including central tendency (e.g. means) or other basic estimates (e.g. regression coefficient) AND variation (e.g. standard deviation) or associated estimates of uncertainty (e.g. confidence intervals)
☐	☒	For null hypothesis testing, the test statistic (e.g. *F*, *t*, *r*) with confidence intervals, effect sizes, degrees of freedom and *P* value noted<br>*Give P values as exact values whenever suitable.*
☒	☐	For Bayesian analysis, information on the choice of priors and Markov chain Monte Carlo settings
☐	☒	For hierarchical and complex designs, identification of the appropriate level for tests and full reporting of outcomes
☒	☐	Estimates of effect sizes (e.g. Cohen's *d*, Pearson's *r*), indicating how they were calculated

Our web collection on statistics for biologists contains articles on many of the points above.

Software and code

Policy information about availability of computer code

Data collection	Independent NeuroScience Services two-photon microscope equipped with Ti:sapphire laser (Chameleon Vision S, Coherent), resonant scanning system (CRS08K, Cambridge Technology), photomultiplier tubes (PMT2102, Thorlabs) and ScanImage software (Versin 5.7, Vidrio. Technologies, MATLAB 2019b) were used to acquire two-photon images. Behavioral experiments were performed by using RZ6 (Tucker Davis Technologies), NI USB-6008 (National Instruments) and MATLAB2020a (MathWorks).
Data analysis	Imaging data were first processed with Suite2p (Pachitariu et al., 2017, v0.10.3) and then analyzed using MATLAB2020a (MathWorks) and Prism9. Custom-written code to generate figures and supplementary figures has been deposited in the German Neuroinformatics Node (https://doi.org/10.12751/g-node.1rfzbn).

For manuscripts utilizing custom algorithms or software that are central to the research but not yet described in published literature, software must be made available to editors and reviewers. We strongly encourage code deposition in a community repository (e.g. GitHub). See the Nature Portfolio guidelines for submitting code & software for further information.

Data

Policy information about availability of data

All manuscripts must include a data availability statement. This statement should provide the following information, where applicable:

- Accession codes, unique identifiers, or web links for publicly available datasets
- A description of any restrictions on data availability
- For clinical datasets or third party data, please ensure that the statement adheres to our policy

Data to generate Figs. 1-4 and Supplementary Figs. 1-19 of this paper can be accessed in Source Data Main and Supplement, respectively. Statistical results are shown in Supplementary Table 1. The data generated in this study has been deposited in the German Neuroinformatics Node (https://doi.org/10.12751/g-node.7xxnmw).

Research involving human participants, their data, or biological material

Policy information about studies with human participants or human data. See also policy information about sex, gender (identity/presentation), and sexual orientation and race, ethnicity and racism.

Reporting on sex and gender

Use the terms sex (biological attribute) and gender (shaped by social and cultural circumstances) carefully in order to avoid confusing both terms. Indicate if findings apply to only one sex or gender; describe whether sex and gender were considered in study design; whether sex and/or gender was determined based on self-reporting or assigned and methods used. Provide in the source data disaggregated sex and gender data, where this information has been collected, and if consent has been obtained for sharing of individual-level data; provide overall numbers in this Reporting Summary. Please state if this information has not been collected. Report sex- and gender-based analyses where performed, justify reasons for lack of sex- and gender-based analysis.

Reporting on race, ethnicity, or other socially relevant groupings

Please specify the socially constructed or socially relevant categorization variable(s) used in your manuscript and explain why they were used. Please note that such variables should not be used as proxies for other socially constructed/relevant variables (for example, race or ethnicity should not be used as a proxy for socioeconomic status). Provide clear definitions of the relevant terms used, how they were provided (by the participants/respondents, the researchers, or third parties), and the method(s) used to classify people into the different categories (e.g. self-report, census or administrative data, social media data, etc.) Please provide details about how you controlled for confounding variables in your analyses.

Population characteristics

Describe the covariate-relevant population characteristics of the human research participants (e.g. age, genotypic information, past and current diagnosis and treatment categories). If you filled out the behavioural & social sciences study design questions and have nothing to add here, write "See above."

Recruitment

Describe how participants were recruited. Outline any potential self-selection bias or other biases that may be present and how these are likely to impact results.

Ethics oversight

Identify the organization(s) that approved the study protocol.

Note that full information on the approval of the study protocol must also be provided in the manuscript.

Field-specific reporting

Please select the one below that is the best fit for your research. If you are not sure, read the appropriate sections before making your selection.

☒ Life sciences ☐ Behavioural & social sciences ☐ Ecological, evolutionary & environmental sciences

For a reference copy of the document with all sections, see nature.com/documents/nr-reporting-summary-flat.pdf

Life sciences study design

All studies must disclose on these points even when the disclosure is negative.

Sample size

Sample sizes of experiments are based on standards in the field.

Data exclusions

Mice were trained for a maximum of 28 sessions. Due to slower learning, one out of eight mice reached only two reversal expert sessions before the training had to be terminated. Furthermore, in two out of eight mice the training had to be briefly suspended due to technical issues, which did not have a major impact on task performance after re-initiation of the paradigm. In one mouse, an imaging artifact gradually appeared from the lateral edges of the two-photon image in one session. Data from the later stage of this session was excluded from analysis. In one mouse, the data recording of one session was aborted in the middle of the session due to the malfunction of the acquisition system. Since the number of trials reached more than half of the session, the data obtained in this session was included for the data analyses.

Replication

Findings were replicated across 8 mice.

Randomization

Provided in Materials and Methods. No formal randomization. Mice were trained counterbalanced in the Go/Nogo reversal learning task.

Blinding	Blinding was not applied for automated behavioral training and analysis and would not be possible due to the nature of the stimulus presentation and their reward contingencies.

Reporting for specific materials, systems and methods

We require information from authors about some types of materials, experimental systems and methods used in many studies. Here, indicate whether each material, system or method listed is relevant to your study. If you are not sure if a list item applies to your research, read the appropriate section before selecting a response.

Materials & experimental systems

n/a	Involved in the study	
☐	☒	Antibodies
☒	☐	Eukaryotic cell lines
☒	☐	Palaeontology and archaeology
☐	☒	Animals and other organisms
☒	☐	Clinical data
☒	☐	Dual use research of concern
☒	☐	Plants

Methods

n/a	Involved in the study	
☒	☐	ChIP-seq
☒	☐	Flow cytometry
☒	☐	MRI-based neuroimaging

Antibodies

Antibodies used	Provided in Materials and Methods, Histology. Primary antibody: Goat anti-calretinin, 1:1000, CG1, Swant and secondary antibodies: Donkey anti-goat 647, 1:1000, A-21447, ThermoFisher
Validation	Commercial antibodies were validated by manufacturers.

Animals and other research organisms

Policy information about studies involving animals; ARRIVE guidelines recommended for reporting animal research, and Sex and Gender in Research

Laboratory animals	Provided in Materials and Methods. Six to 10-week-old (at the start of the experiment) male C57BL/6J mice were used throughout this study.
Wild animals	The study did not involve wild animals.
Reporting on sex	Only male mice were used in this study
Field-collected samples	The study did not involve animals collected from the field.
Ethics oversight	Experiments were performed in accordance with the institutional guidelines of the University of Basel (University of Basel, Tierschutz) and DZNE, Bonn and were approved by the Cantonal Veterinary Office of Basel-Stadt, Switzerland or the Landesamt für Natur-Umwelt- und Verbraucherschutz Nordrhein-Westfalen, Germany.

Note that full information on the approval of the study protocol must also be provided in the manuscript.
